# Increased levels of midbrain immune-related transcripts in schizophrenia and in murine offspring after maternal immune activation

**DOI:** 10.1038/s41380-019-0434-0

**Published:** 2019-06-05

**Authors:** Tertia D. Purves-Tyson, Ulrike Weber-Stadlbauer, Juliet Richetto, Debora A Rothmond, Marie A. Labouesse, Marcello Polesel, Kate Robinson, Cynthia Shannon Weickert, Urs Meyer

**Affiliations:** 1 Schizophrenia Research Laboratory, Neuroscience Research Australia, Sydney, NSW 2031 Australia; 2 School of Psychiatry, University of New South Wales, Sydney, NSW 2052 Australia; 3 Institute of Pharmacology and Toxicology, University of Zurich-Vetsuisse, Zurich, Switzerland; 4 Physiology and Behavior Laboratory, ETH Zurich, Schwerzenbach, Switzerland; 5 Department of Psychiatry, College of Physicians and Surgeons, Columbia University, 1051 Riverside Drive, NYC, 10032 NY USA; 6 Institute of Anatomy, University of Zurich, Zurich, Switzerland; 7 Department of Neuroscience and Physiology, Upstate Medical University, Syracuse, 13210 New York USA; 8 Neuroscience Centre Zurich, University of Zurich and ETH Zurich, Zurich, Switzerland

**Keywords:** Schizophrenia, Neuroscience, Molecular biology, Schizophrenia, Neuroscience

## Abstract

The pathophysiology of dopamine dysregulation in schizophrenia involves alterations at the ventral midbrain level. Given that inflammatory mediators such as cytokines influence the functional properties of midbrain dopamine neurons, midbrain inflammation may play a role in schizophrenia by contributing to presynaptic dopamine abnormalities. Thus, we quantified inflammatory markers in dopaminergic areas of the midbrain of people with schizophrenia and matched controls. We also measured these markers in midbrain of mice exposed to maternal immune activation (MIA) during pregnancy, an established risk factor for schizophrenia and other psychiatric disorders. We found diagnostic increases in SERPINA3, TNFα, IL1β, IL6, and IL6ST transcripts in schizophrenia compared with controls (*p* < 0.02–0.001). The diagnostic differences in these immune markers were accounted for by a subgroup of schizophrenia cases (~ 45%, 13/28) showing high immune status. Consistent with the human cohort, we identified increased expression of immune markers in the midbrain of adult MIA offspring (SERPINA3, TNFα, and IL1β mRNAs, all *p* ≤ 0.01), which was driven by a subset of MIA offspring (~ 40%, 13/32) with high immune status. There were no diagnostic (human cohort) or group-wise (mouse cohort) differences in cellular markers indexing the density and/or morphology of microglia or astrocytes, but an increase in the transcription of microglial and astrocytic markers in schizophrenia cases and MIA offspring with high inflammation. These data demonstrate that immune-related changes in schizophrenia extend to dopaminergic areas of the midbrain and exist in the absence of changes in microglial cell number, but with putative evidence of microglial and astrocytic activation in the high immune subgroup. MIA may be one of the contributing factors underlying persistent neuroimmune changes in the midbrain of people with schizophrenia.

## Introduction

Increasing evidence suggests that the immune system is involved in the pathogenesis and pathophysiology of schizophrenia. Support for this notion includes epidemiological findings of increased risk of schizophrenia following early-life exposure to infectious pathogens or inflammatory stimuli [1–3], along with postmortem and imaging studies demonstrating glial anomalies [4–15] and increased expression of cytokines and other mediators of inflammation in the brain and periphery in people with schizophrenia [4–7, 16–21]. Noticeable inflammatory abnormalities, however, are evident only in a subgroup of schizophrenia cases [4–6, 17] and may predict poorer clinical outcomes and treatment responses [18, 20].

One of the most recognized pathophysiological features of schizophrenia is dopamine dysregulation [22, 23], which includes dopaminergic alterations at the level of the ventral midbrain [24, 25]. The ventral midbrain contains the majority of dopamine cell bodies that give rise to the mesolimbic, mesocortical, and nigrostriatal dopamine pathways [26]. Inflammatory mediators such as cytokines and chemokines influence the development, maintenance, and functional properties of midbrain dopamine neurons [27–30]. Pro-inflammatory cytokines such as interleukin (IL)-1β promote the differentiation of mesencephalic progenitor cells into dopaminergic neurons [27], and IL6 modulates dopaminergic activity in various mesolimbic structures in a dose-dependent manner [29, 30]. Abnormal inflammatory processes in the midbrain may thus have a role in schizophrenia by contributing to presynaptic dopamine abnormalities [24, 25]. Against these backgrounds, we hypothesized that people with schizophrenia would have increased expression of immune-related markers in the dopaminergic region of the midbrain.

We tested this hypothesis by measuring transcripts of several pro-inflammatory cytokines and the acute-phase protein, serpin family A member 3 (SERPINA3), in postmortem tissue encompassing the substantia nigra of schizophrenia cases and matched controls. IL1β, IL6, IL8, IL18, tumor necrosis factor (TNF)-α, IL6 signal transducer (IL6ST), IL1A, IL17RA, and SERPINA3 mRNA levels were measured using quantitative polymerase chain reaction (qPCR). Given that microglia and astrocytes are the main immune-competent cells in the brain parenchyma and secrete inflammatory cytokines [31, 32], we measured gene expression of the microglia markers, allograft inflammatory factor 1 (AIF1, also known as IBA1) and cluster of differentiation 68 (CD68). We also assessed the number of cells expressing human leukocyte antigen (HLA), which include microglia, in the substantia nigra of cases and controls. In addition, we measured translocator protein (TSPO) gene expression, which is expressed on microglia and other cell types and has often been used as a surrogate microglial marker in imaging studies [33]. Finally, we also measured gene expression of the astrocyte marker, glial fibrillary acidic protein (GFAP) to index astrocytic activity. These investigations provide the first attempt to identify possible neuroimmune changes in a brain area that pertains to presynaptic dopamine abnormalities in schizophrenia.

Postmortem studies are, however, limited in their capacity to elucidate the etiology of the anticipated neuroimmune changes, particularly owing to the possible confounds of antipsychotic treatment in cases with schizophrenia. To address this limitation, we investigated whether similar abnormalities could be detected in a mouse model that has etiological, pathophysiological, and symptomatological relevance for schizophrenia and related disorders. We used the maternal immune activation (MIA) model [2, 34, 35], which was developed based on epidemiological evidence linking MIA with increased risk of schizophrenia and related disorders in the offspring [1, 3]. MIA was induced by the viral mimic polyriboinosinic–polyribocytidylic acid [poly(I:C)], which induces a cytokine-associated acute-phase response in maternal and fetal compartments [2, 34, 35]. Importantly, MIA in rodents leads to numerous schizophrenia-relevant disturbances in the offspring [2, 34, 35], including deficits in sensorimotor gating, impairments in selective attention, and increased sensitivity to amphetamine [36–39], all of which involve dopaminergic neurotransmission [40, 41]. To complement the postmortem investigations in our human cohort, we measured gene expression of a similar array of pro-inflammatory and acute-phase proteins in the substantia nigra of adult MIA offspring and controls, and assessed the density of microglia expressing AIF1 and CD68 and astrocytes expressing GFAP by immunohistochemistry in this dopamine-rich brain area. Given that morphological changes are often used to index microglia activity [31], we also measured soma size, number of primary processes and number of process branch points of the AIF-positive microglia. Hence, the MIA model allowed us (1) to evaluate whether early-life exposure to immune activation may represent an etiological factor for lasting neuroimmune changes in the midbrain, and (2) to identify possible immune-related gene expression changes in a model that captures schizophrenia-relevant pathophysiological and behavioral abnormalities without the confound of antipsychotic medication.

We hypothesized that the transcripts of immune markers and immune-related cells will be increased in the midbrain of people with schizophrenia and of adult mice that were exposed to MIA. Based on our recent stratification studies, [4–6, 16] we further hypothesized that a subset of schizophrenia cases will have immune transcript levels well above controls, and that the proportion of cases in this elevated subgroup would be increased in people with schizophrenia and in adult mice that were exposed to MIA.

## Methods

### Human postmortem tissue collection and cohort demographics

Experiments involving human tissue were approved by the University of New South Wales Human Research Ethics Committees (HREC12435). The midbrain tissue, neuroanatomically matched at the level of the oculomotor nerve (28 schizophrenia [42]; 29 controls) was collected as previously described [NSWs Tissue Resource Centre (Sydney, Australia)] [25]. Sample size was chosen based on previous postmortem studies (minimum 25 cases required to detect a 1.25-fold change, 80% power, *α* = 0.05) [42]. Substantia nigra (ventral to the red nucleus) was excised for RNA extraction from six cryostat-generated 60 μm slices based on the region outlined from tyrosine hydroxylase immunolabelling of adjacent 14-μm slide-mounted sections [25].

Age, postmortem interval (PMI), and RNA integrity number (RIN) did not differ significantly between diagnostic groups, but pH was lower in schizophrenia [25, 43] (see demographics, Table 1). All patients received antipsychotic medication [25] converted to chlorpromazine (CPZ) equivalents (lifetime, daily, and last dose, Table 1) [44, 45]. Details of the midbrain postmortem cohort were previously published [25] and further details including cause of death, effect of antipsychotics (first generation vs. second generation, treatment resistant vs. treatment responsive), depression, suicide, mode of death, and smoking are also provided in the Supplementary Information.

**Table 1:** Demographic and clinical details of postmortem midbrain mRNA cohort

	mRNA cohort		
Demographic	Control mRNA (*n* = 29)	Schizophrenia mRNA (*n* = 28)	Statistics mRNA
Age (years)	51.2 (22–69)	51.4 (26–67)	*p* = 0.90, df = 55, t = −0.06
Gender (M, F)	(20,9)	(19,9)	–
pH	6.67 ± 0.26	6.51 ± 0.20*	*p* = 0.01, df = 55, t = 2.6
PMI h	31.9 ± 10.12 (15–50)	35.7 ± 17.71 (5–72)	*p* = 0.33, df = 55, t = −0.98
RIN	5.6 ± 1.14	5.6 ± 1.31	*p* = 0.95, df = 55, t = −0.06
Duration of illness (years)	na	28.31 ± 12.72 (4–49) [28, 19–39]	–
Daily chlorpromazine equivalent dose (mg)	na	736.45 ± 520.50 (*n* = 22) [583.0, 387.5–825.0]	–
Last recorded chlorpromazine equivalent dose (mg)	na	597.54 ± 497.64 (*n* = 28) [462.5, 192.5–927.0]	–
Lifetime chlorpromazine equivalent dose (g)	na	8231.44 ± 8714.24 (*n* = 22) [4726.8, 3442.0–8563.0]	–
Depression symptoms over lifetime (yes, no, unknown)	1 (SSRI), 26, 2	8 (6 on SSRIs, 2 on TCAs), 19, 1	–
Symptoms (mostly positive, mostly negative, unknown)	na	18, 7, 3	–
Agonal state (rapid/intermediate/prolonged/unknown)	18/10/0/1	17/9/0/2	–
Manner of death^$^ (illness, trauma, natural causes/suicide)	29/0	21/7	
Smoker at time of death (yes/no/unknown)	11/12/6	16/6/6	–

*F* female, *M* male, *RIN* RNA integrity, *PMI* postmortem interval, *na* not applicable, *SSRIs* serotonin reuptake inhibitors, *TCAs* tricyclic antidepressants

Data are mean ± SD (range) (median, interquartile range). ^$^See Supplementary Material for further information. *p < 0.05

### MIA model

Detailed information regarding the MIA model can be found in the Supplementary Information. In brief, female C57BL6/N mice (Charles Rivers, Sulzfeld, Germany) were time-mated as previously described [38]. Pregnant dams were given either a single injection of poly(I:C) (potassium salt; Sigma–Aldrich, Buchs, St Gallen, Switzerland) (*n* = 13) or vehicle (*n* = 14) on gestational day (GD) 17. GD17 in the mouse roughly corresponds to human gestational weeks 13–14 in terms of midbrain development (http://translatingtime.org/translate). It was selected because of our previous immunohistochemical and imaging studies showing dopamine-related cellular and volumetric changes in the ventral midbrain of adult offspring exposed to MIA at GD17 [46, 47]. Poly(I:C) (5 mg/kg) was dissolved in 0.9% NaCl (vehicle) and was administered intravenously into the tail vein (final concentration, 1 mg/ml). Offspring were weaned on postnatal day (PND) 21 and kept until adulthood (PND 120). To minimize possible litter effects and confounds arising from technical replicates [48], 1–2 male and female offspring were randomly selected from each litter, resulting in a group size of *n* = 32 (16/16 males/females) per group for the transcriptomic analyses and *n* = 10 (5/5 males/females) per group for immunohistochemical analyses. All procedures involving animal experimentation were approved by the Cantonal Veterinarian’s Office of Zurich (approval nr. ZH 172/2015).

### RNA extraction and quantitative real-time PCR

Total RNA was extracted from human substantia nigra using Trizol and cDNA synthesized using Superscript III (Life Technologies, Scoresby, Australia) [25]. RNA quality was determined using the Agilent Bioanalyzer 2100 (Agilent Technologies, Santa Clara, CA) and cases with low RINs (< 4.0) were excluded (one control, two schizophrenia). Quantitative PCR was conducted with the Applied Biosystems Prism 7900HT Fast Real Time system.

Mouse substantia nigra RNA was extracted using RNeasy Plus Universal Mini Kits (Qiagen, Hilden, Germany) according to manufacturer’s instruction. Quantitative PCR was conducted with iScript one-step RT-PCR kits and a Taqman real-time system (CFX384, Bio-Rad Laboratories, Cressier, Switzerland) as previously [47, 49].

Human and mouse TaqMan gene expression assays were used (Life Technologies, Australia, and Zug, Switzerland, respectively; Supplementary Table 1). Housekeeper controls (β-actin, Tata-binding protein, ubiquitin-C mRNAs) and the geomean of all three did not differ according to diagnosis (all *t* < 0.74, *df* = 55, *p* > 0.05) in the human cohort [25]. In the MIA model, ribosomal phosphoprotein (36B4) was used as the housekeeper control as validated previously [47, 49]. 36B4 expression was unaffected by MIA (*t* < 0.65, *df* = 62, *p* > 0.05). Relative gene expression was calculated with the 2^−ΔΔCt^ method [50], normalizing to 36B4 (mouse) [47, 49] or housekeeper Ct geomean (human) [51]. Gene expression data are presented as fold change of relative mRNA levels ± SEM.

### Mouse cytokine protein measurements

Cytokine proteins (IL6, TNFα, and IL1β) in midbrain homogenates were quantified using a customized Meso-Scale Discovery V-Plex electrochemiluminescence assay for mice. See Supplementary Information for details.

### Quantification of microglia and astrocytes by immunohistochemistry

3,3′-diaminobenzidine immunohistochemistry with antibodies against microglial markers was used [4, 52]. Frozen human midbrain sections (14 μm) on glass slides were incubated with an HLA-DR antibody (M0775; Dako, North Sydney, NSW, Australia) and a horse anti-mouse biotinylated secondary antibody (BA-2000; Vector Laboratories). The HLA-DP/DR/DQ gene encodes subunits of the MHC-II receptor expressed in antigen-presenting cells, including but not limited to microglia. HLA-DR-positive (HLA+) cells were counted in two dimensions in the human substantia nigra (ventral to the red nucleus and based on tyrosine hydroxylase staining of adjacent sections) and calculated as total number of HLA+ cells divided by the total area counted (0.2353 mm^2^) and cell density expressed as cells/mm^2^ (for detail see also Supplementary Information).

In the MIA model, mice were perfused intracardially with 4% phosphate-buffered paraformaldehyde (PFA) that contained 15% picric acid, followed by post-fixation in PFA and cryoprotection [50]. The mouse brains were cut coronally with a sliding microtome (30 μm, eight serial sections) and immunohistochemistry was performed on floating coronal sections using rabbit anti-AIF1 (019–19741; Wako, Neuss, Germany), rat anti-CD68 (MCA1957GA; Serotec, Oxford, UK), or mouse monoclonal anti-GFAP (MAB360; EMD Millipore, Billerica, USA) [52]. Total AIF1+, CD68+, and GFAP+ cells were quantified in the mouse substantia nigra in three dimensions using stereological estimations [53] and expressed as cells/mm^3^ (for detail, see also Supplementary Information).

### Human endothelial cell culture and antipsychotic treatment

Human endothelial cells hCMEC/D3 [54] were cultured and treated with antipsychotics as previously described [55]. In brief, confluent cells were treated with 1.2 μm clozapine (Abcam, Cambridge, UK), 26.6 nm haloperidol (Abcam), 0.974 μm Risperidone (Abcam) (based on typical therapeutic serum ranges) or vehicle (5% fetal bovine serum) for 48 h before harvesting the cells for RNA extraction and cDNA synthesis and qPCR.

### Statistical analyses

SPSS (v23, IBM, Armonk, NY) and Prism (v6, GraphPad, La Jolla, CA) were used, with significance set at *p* ≤ 0.05. A detailed description of the statistical analyses and clustering method [4–6, 16] is in the Supplementary Information.

## Results

### Midbrain immune transcripts are increased in schizophrenia and in MIA offspring

**Fig. 1:**
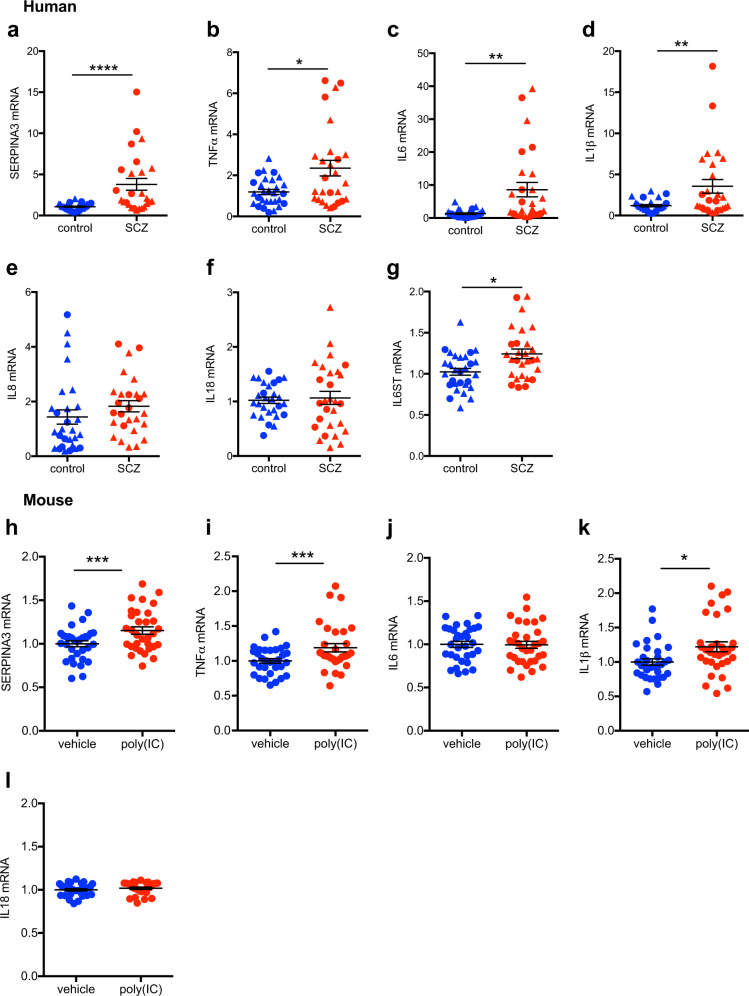
Midbrain inflammatory mRNA transcripts in schizophrenia (SCZ, **a**–**g**) and in adult mice prenatally exposed to the viral mimetic, poly(I:C) **h**–**l**. The scatter plots depict relative mRNA levels by diagnosis (human cohort: SCZ versus control subjects, circles represent women, triangles represent men) and by prenatal treatment group (mouse cohort: poly(I:C) versus vehicle controls, for analysis by sex, see Supplementary Fig. 2). Human cohort: **a** SERPINA3, **b** TNFα, **c** IL6, **d** IL1β, **e** IL8, **f** IL18, and **g** IL6ST mRNA. Mouse cohort: **h** SERPINA3, **i** TNFα, **j** IL6, **k** IL18, and **l** IL1β mRNA. Data are mean ± SEM. *p < 0.05, **p < 0.01, ****p < 0.0001

In support of our hypothesis, we found that transcripts of pro-inflammatory and acute-phase markers were upregulated in the midbrain of people with schizophrenia compared with matched controls. SERPINA3 mRNA was increased by 414% (*F* = 21.49, *df* = 1,52, *p* < 0.0001; Fig. 1a) in schizophrenia cases relative to controls and TNFα mRNA was increased by 132% (*F* = 3.90, *df* = 1,53, *p* = 0.050) in cases compared with controls (Fig. 1b). IL6 (*F* = 11.789, *df* = 1,52, *p* = 0.001; Fig. 1c) and IL1β (*t* = −2.784, *df* = 41,22, *p* = 0.001; Fig. 1d) mRNAs were increased by 557% and 261%, respectively. A modest but statistically significant (*F* = 6.114, *df* = 1,53, *p* = 0.017) increase was found for IL6ST mRNA in people with schizophrenia compared with controls (Fig. 1g), whereas IL8 and IL18 mRNAs (both *F* < 1.0, df = 1,52/53, *p* > 0.05) were unchanged (Fig. 1e, f).

Consistent with the human cohort, we identified increased transcripts of immune markers in the midbrain of MIA-exposed mice relative to controls. Specifically, we found SERPINA3 (*F* = 8.392, *df* = 60,1, *p* = 0.005; Fig. 1h), TNFα (*F* = 9.238, *df* = 56,1, *p* = 0.003; Fig. 1i) and IL1β (*F* = 7.178, *df* = 1,60, *p* = 0.01; Fig. 1k) mRNAs to be increased in MIA-exposed relative to vehicle-exposed offspring. Some of these effects were influenced by the sex of the offspring (Supplementary Fig. 1). IL6 (Fig. 1j) and IL18 (Fig. 1l) mRNAs were not changed by MIA compared with prenatal control treatment (both *F* < 0.10, *df* = 1,56, *p* > 0.05), but were influenced by sex only (see Supplementary Fig. 1).

Given that the effect sizes of cytokine gene expression changes in the MIA model were relatively modest, we performed additional measurements of cytokine proteins in the midbrain of MIA-exposed and control mice. Consistent with the gene expression analyses, MIA-exposed offspring displayed a significant increase in midbrain TNFα (main effect of prenatal treatment: *F* = 12.72, *df* = 20,1, *p* = 0.002) and IL1β (main effect of prenatal treatment: *F* = 5.56, *df* = 20,1, *p* = 0.029) protein levels, whereas midbrain IL6 protein levels were not affected by MIA (*F* < 0.04) (Refer to Supplementary Information and Supplementary Fig. 2).

### Midbrain microglia and astrocyte markers in schizophrenia and in MIA offspring

**Fig. 2:**
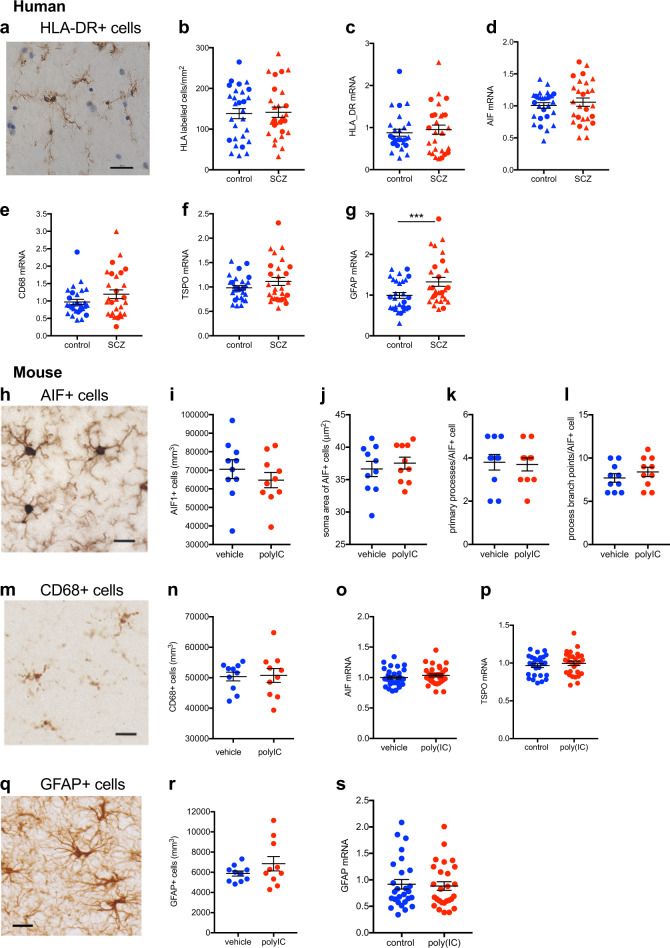
Midbrain microglia and astrocyte markers and microglial and astrocytic cell density in schizophrenia (SCZ) and in adult mice prenatally exposed to the viral mimetic, poly(I:C). **a** Representative immunohistochemical staining showing microglial cells in the human midbrain, which were identified by brown HLA+ staining in the cytoplasm and processes. Nuclei are stained blue with a nuclear dye (Nissl). **b** HLA-DR+ cell number in midbrain from SCZ cases compared with control cases. **c** HLA-DR, **d** AIF1, **e** CD68 **f** TSPO, and **g** GFAP mRNA levels in midbrain from SCZ cases compared with control cases. Representative immunohistochemical staining of **h** AIF1+ and **m** CD68+ microglial cells and **q** GFAP+ astrocytes in mouse midbrain. **i** AIF1+ and **n** CD68+ microglial cell density and **r** GFAP+ astrocyte cell density in midbrain from adult mice prenatally exposed to poly(I:C) or vehicle control. AIF+ microglial cell **j** soma area, **k** primary processes, and **l** process branch points in midbrain from adult mice prenatally exposed to poly(I:C) or vehicle control. **o** AIF1 **p** TSPO, and **s** GFAP mRNA levels in midbrain from adult mice prenatally exposed to poly(I:C) or vehicle control. Data are mean ± SEM. Scale bars = 20 μm

Despite the alterations in transcript levels of immune markers (Fig. 1), we found no evidence of changes in microglial cell numbers in the midbrain of schizophrenia cases relative to controls (Fig. 2). The number of HLA-DR+ cells (Fig. 2a) correlated positively with age at death in schizophrenia (*r* = 0.46, *p* = 0.016, *N* = 27), but not in control cases (*r* = 0.225, *p* = 0.249, *N* = 28). HLA-DR+ cell number, however, did not differ in the midbrain between schizophrenia and controls when co-varying with age (*F* = 0.046, *df* = 54, *p* = 0.845; Fig. 2b). In agreement with the immunohistochemical data, HLA-DR, AIF1, TSPO, and CD68 mRNA levels in the midbrain did not differ between people with schizophrenia and controls (all *F* = 2.134, *df* = 1,54, *p* = 0.15; Fig. 2c–f). There was, however, a significant increase in gene expression of the astrocyte marker, GFAP, in the midbrain of schizophrenia cases relative to controls (*F* = 11.034, *df* = 1,53, *p* = 0.002) (Fig. 2g).

Consistent with the human cohort, neither AIF1+ (Fig. 2h, i) nor CD68+ (Fig. 2m, n) cell densities (both *F* < 0.65, *df* = 3,16, *p* > 0.05) were altered in the midbrain of MIA-exposed mice compared with controls. Morphological measures (soma size, number of primary processes, and process branch points) of AIF+ microglia were also not changed in the midbrain of MIA-exposed mice compared with controls (all *F* < 0.90, *df* = 1,16, *p* > 0.05; Fig. 2j–l). Likewise, midbrain AIF1 and TSPO mRNA levels were not different between MIA-exposed offspring and controls (both *F* < 1.0, *df* = 1,60/51, *p* > 0.05; Fig. 2o, p). In contrast to the human cohort, GFAP mRNA was unchanged between MIA-exposed offspring and controls (*F* = 0.379, *df* = 1,51, *p* = 0.541; Fig. 2s) and GFAP+ cell density (*F* = 1.46, *df* = 1,16, *p* > 0.05; Fig. 2q) was not altered between MIA-exposed offspring and controls. There were no main effects of sex or sex × treatment interactions (all *F* < 1.2, *df* = 1,51–60, *p* > 0.05) in terms of AIF1, TSPO, and GFAP mRNA levels or AIF+, CD68+, or GFAP+ cell density (Supplementary Fig. 3).

### A subgroup of schizophrenia cases show elevated midbrain immune markers

**Fig. 3:**
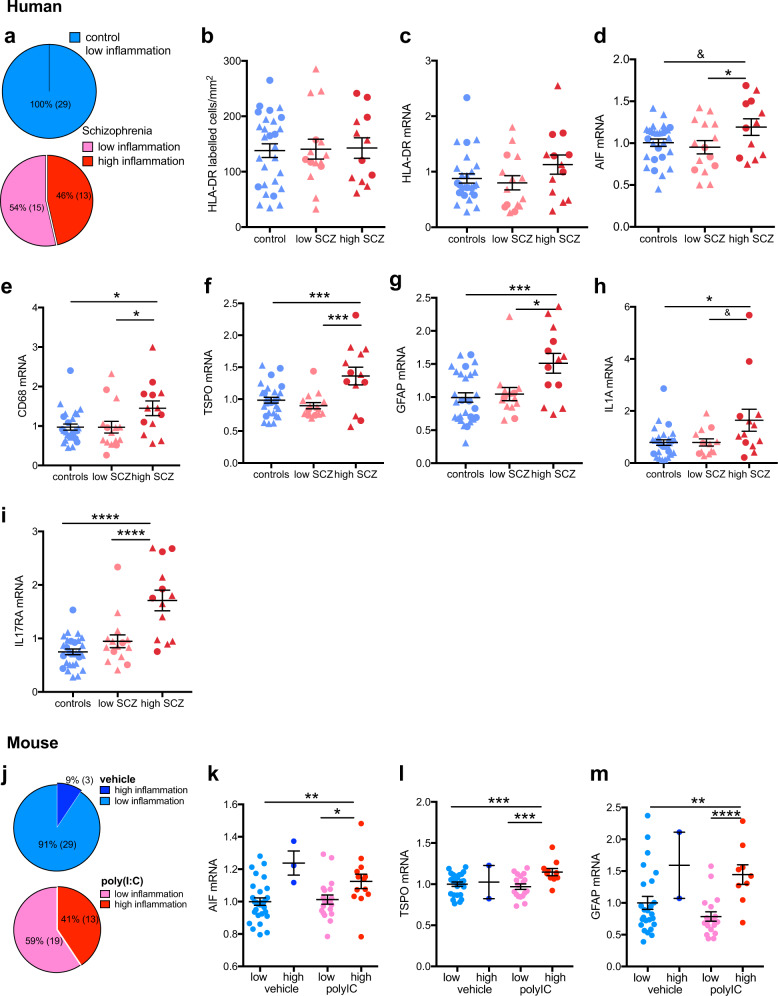
Changes in immune-related mRNA expression in midbrain from schizophrenia (SCZ) and control cases and from adult mice prenatally exposed to the viral mimetic, poly(I:C), after stratification into “high” and “low” immune subgroups. **a** Cluster analysis of immune-related gene expression in midbrain from schizophrenia (SCZ) and control cases revealed that all control cases (blue) and 54% of schizophrenia cases (pink) were of low immune status, whereas 46% of SCZ cases were classified as high immune. **b** HLA+ microglial cell number and **c** HLA-DR mRNA did not change in the immune subgroups. **d** AIF1 mRNA was increased in the SCZ/high immune compared to the SCZ/low immune subgroup. **e** CD68 and **f** TSPO mRNAs were increased in the SCZ/high subgroup compared with the SCZ/low and the control groups. **g** GFAP mRNA was increased in the SCZ/high subgroup compared with the SCZ/low and the control groups. IL1A **h** and IL17RA **i** mRNAs were increased in SCZ/high compared with controls and IL17RA mRNA was increased in SCZ/high compared with the SCZ/low subgroup. **j** Cluster analysis of immune-related gene expression in midbrain from adult mice prenatally exposed to the viral mimetic, poly(I:C) revealed that 91% of vehicle–offspring were of low immune status (light blue) and 9% were of immune status (dark blue). Among poly(IC)-exposed offspring, 59% were classified as low immune (pink), whereas 41% were classified as high immune (red). **k** AIF1, **l** TSPO, and **m** GFAP mRNAs were increased in the poly(IC)/high immune compared with the poly(IC)/low immune and vehicle/low immune subgroups. Data are mean ± SEM. ^&^*p* < 0.1, *p < 0.05, ***p < 0.001, ****p < 0.0001

In keeping with our previous efforts to define subgroups of schizophrenia cases with low and high immune status in cortical tissue and blood [4–6], we performed a two-step cluster analysis of immune-related gene expression in the entire (cases and controls) human cohort. This analysis revealed 13 (22.8%) individuals in a high immune cluster and 44 (77.2%) individuals in a low immune cluster. The high immune subgroup was defined by high SERPINA3, IL6, IL1β, and TNFα mRNAs (all *t* > 5.0, *p* < 0.001 between high and low immune groups). The 13 cases in the high immune subgroup were all schizophrenia cases (schizophrenia/high immune) (Fig. 3a). Hence, the remaining 15 schizophrenia cases had low inflammatory markers (schizophrenia/low immune), as did all control cases (*n* = 29; control/low immune) (Figure 3a) (*χ*^2^ = 57.0, *P* < 0.0001, *N* = 57). There was an equivalent distribution of males and females between the diagnosis/immune groups (*χ*^2^ = 0.457, *p* < 0.796, *N* = 57). Running the cluster algorithm 20 times on a subset of cases (*n*−3) showed that 88% (48/54) of cases remained in the same cluster 100% of the time. Of the 12% (6/54) of cases that switched cluster, two cases remained in the same cluster 75% of the time, two cases remained in the same cluster 70% of the time and one case remained in the same cluster 55% of the time.

HLA-DR+ cell numbers (cells/mm^2^) and immune marker gene expression were compared between the diagnosis/immune groups. HLA-DR+ cell density in the SN and HLA-DR mRNA were unchanged between subgroups (*F* < 0.2, *df* = 2,51, *p* > 0.05; Fig. 3b, c). However, AIF1, CD68, and TSPO mRNA levels were significantly different according to subgroup (all *F* > 3.84, *df* = 2,51–53, *p* < 0.05; Fig. 3d–f) with increased AIF1, CD68 and TSPO mRNA in the schizophrenia/high immune subgroup compared with the schizophrenia/low immune subgroup (*p* < 0.05) and in the schizophrenia/high immune subgroup compared with the control group for CD68 and TSPO mRNAs (*p* < 0.05). GFAP mRNA was also increased in the schizophrenia/high immune subgroup compared with the schizophrenia/low immune and control subgroups (*p* < 0.05) (Fig. 3g).

As expected, the immune transcripts that contributed to the high and low immune groups (SERPINA3, TNFα, IL6, IL1β) were elevated in the schizophrenia/high immune subgroup above both the control/low and the schizophrenia/low immune subgroups (see Supplementary Fig. 4 to view magnitude of change). Of the markers that did not contribute to the final cluster, IL8 and IL18 mRNAs were not significantly different between the subgroups, whereas IL6ST was increased in both the schizophrenia/high and schizophrenia/low immune subgroups compared with the control/low immune subgroup (Supplementary Fig. 4).

We analyzed two further immune-related markers, IL1A and IL17RA, not used to generate the cluster. By diagnosis, there was a trend toward an increase of IL1A mRNA in schizophrenia cases (*t* = −1.731, *df* = 52, *p* = 0.089) and IL17RA mRNA was significantly increased in schizophrenia cases compared with control subjects (*t* = −3.952, *df* = 54, *p* < 0.001) (data not shown). However, both IL1A (Figure 3h) (*F* = 3.353, *df* = 2,51, *p* = 0.043) and IL17RA (Figure i) (*F* = 16.543, *df* = 2,53, *p* < 0.0001) mRNAs were elevated in the schizophrenia/high immune subgroup compared with the control/low immune subgroup (108.4% and 128.2%, both *p* < 0.05) and compared with the schizophrenia/low immune subgroup (108.2% and 101.9%; IL1A *p* = 0.065, IL17RA *p* < 0.0001).

### A subgroup of MIA offspring show elevated midbrain immune markers

Cluster analysis of immune gene expression in the MIA model revealed that 16 (25%) mice were part of a high immune subgroup, whereas 48 (75%) were part of a low immune subgroup. The high immune subgroup was defined by high SERPINA3, IL6, IL1β, IL18, and TNFα mRNAs (IL6 *t* = −8.088, *p* = 0.001; SERPINA3, IL1β, IL18, TNFα, all U between 174 and 280, *p* < 0.002). Thirteen mice in the high immune subgroup were poly(I:C)-exposed offspring (poly(I:C)/high immune) and three were control offspring (vehicle/high immune) (Fig. 3j). The remaining 29 control and 19 poly(I:C)-exposed offspring were defined as being in the low immune subgroups (vehicle/low immune; poly(I:C)/low immune, respectively) (*χ*^2^ = 8.33, *p* = 0.004, *N* = 64; Fig. 3j). There was an equivalent distribution of sexes between the different immune subgroups (*χ*^2^ = 0.333, *p* = 0.564, *N* = 64).

We further identified a significant effect of immune subgroup on the transcript levels of AIF1, TSPO, and GFAP (all *F* > 4.247, *df* = 2,49–58, *p* < 0.05; Fig. 3k–m). AIF1, TSPO, and GFAP mRNAs were increased in the poly(I:C)/high immune subgroup compared with the vehicle/low immune and the poly(I:C)/low immune subgroups (all between 11–66%, *p* < 0.05). As expected, there was a significant effect of immune subgroup on the transcript levels of SERPINA3, TNFα, IL6, and IL1β (Supplementary Fig. 4) in the MIA model.

### Effects of antipsychotics on midbrain inflammatory transcripts by diagnosis and by immune subgroup

Given that antipsychotics can influence inflammatory markers [56, 57] and immune cells [15, 58], we assessed the effects of antipsychotic medication (as converted to CPZ equivalents) on the mRNA transcripts of immune markers. TNFα, IL1β, IL6, IL1A, IL17RA, AIF, and CD68 mRNAs were positively correlated with daily and TNFα, IL6, IL17RA, and CD68 mRNAs with lifetime CPZ exposure (all *r* > 0.5, *p* < 0.05) (Table 2). SERPINA3, IL8, IL18, IL6ST, TSPO, and GFAP mRNAs did not correlate with any CPZ equivalents (all *p* > 0.05) (Table 2).

**Table 2:** Correlations (Spearman’s) between immune marker gene expression and measures of antipsychotic drug treatment and duration of illness

Gene of interest	Chlorpromazine equivalent measure	*n*	*p*	Correlation coefficient
AIF mRNA	Life time	22	0.428	0.178
	Mean daily	22	0.009	0.546**
	Last dose	27	0.843	0.040
	Illness duration	27	0.402	0.168
CD68 mRNA	Life time	22	0.033	0.456*
	Mean daily	22	0.007	0.556**
	Last dose	28	0.882	0.029
	Illness duration	28	0.343	0.186
TSPO mRNA	Life time	22	0.433	0.176
	Mean daily	22	0.085	0.376^&^
	Last dose	28	0.745	−0.064
	Illness duration	28	0.319	0.195
GFAP mRNA	Life time	22	0.343	0.212
	Mean daily	22	0.178	0.298
	Last dose	28	0.731	−0.068
	Illness duration	28	0.620	0.098
TNFα mRNA	Life time	21	0.020	0.503*
	Mean daily	21	0.001	0.649**
	Last dose	27	0.388	0.173
	Illness duration	27	0.084	0.339^&^
SERPINA3 mRNA	Life time	20	0.201	0.299
	Mean daily	20	0.119	0.360
	Last dose	26	0.209	0.255
	Illness duration	26	0.207	0.256
IL1β mRNA	Life time	21	0.175	0.307
	Mean daily	21	0.021	0.499*
	Last dose	27	0.172	0.271
	Illness duration	27	0.680	0.083^&^
IL6 mRNA	Life time	21	0.010	0.547*
	Mean daily	21	0.003	0.618**
	Last dose	27	0.118	0.308
	Illness duration	27	0.475	0.144
IL8 mRNA	Life time	21	0.088	0.381^&^
	Mean daily	21	0.278	0.248
	Last dose	27	0.547	0.121
	Illness duration	27	0.108	0.316
IL18 mRNA	Life time	22	0.936	0.018
	Mean daily	22	0.691	0.090
	Last dose	28	0.798	−0.051
	Illness duration	28	0.517	0.128
IL6ST mRNA	Life time	22	0.575	0.127
	Mean daily	22	0.867	0.038
	Last dose	28	0.280	−0.211
	Illness duration	28	0.773	0.057
IL1A	Life time	20	0.120	0.359
	Mean daily	20	0.003	0.634**
	Last dose	26	0.343	0.194
	Illness duration	26	0.585	−0.112
IL17RA	Life time	22	0.026	0.473*
	Mean daily	22	<0.0001	0.702****
	Last dose	28	0.287	0.209
	Illness duration	28	0.907	0.023

^&^*p* < 0.1, *p < 0.05, **p < 0.01, ***p < 0.001, ****p< 0.0001

When exploring the data by immune subgroups, there was no difference in duration of illness between the schizophrenia/high immune and schizophrenia/low immune subgroups (*t*(25) = −1.318, *p* = 0.199). However, the schizophrenia/high immune subgroup received higher lifetime CPZ, daily CPZ and last CPZ equivalent doses (all *t*(20–26) < − 2.7, *p* < 0.05). We performed additional analyses of antipsychotics (treatment resistance, first- versus second-generation antipsychotics), clinical state (depression and suicide) and the impact of mode of death and being a smoker at the time of death on midbrain inflammatory marker mRNAs in control and schizophrenia groups, and where possible, in the different immune subgroups (see Supplementary Information).

### Effect of antipsychotic treatment on cytokine gene expression in human brain endothelial cells

IL1β gene expression was altered by antipsychotic treatment of cultured human brain endothelial cells (*F* = 3.60, *df* = 3,26, *p* = 0.03). Specifically, IL1β gene expression was decreased by risperidone relative to vehicle-treated cells (*p* < 0.05) but was unchanged by haloperidol and clozapine (both *p* > 0.05). IL6 gene expression in endothelial cultures was not changed by risperidone, haloperidol or clozapine and SERPINA3 gene expression was also not changed by any antipsychotic (both, *F* < 2.10, *df* = 3,26, *p* > 0.05) (Supplementary Fig. 5).

## Discussion

We provide the first evidence of inflammation-related abnormalities in the midbrain of people with schizophrenia relative to matched controls. We found marked diagnostic increases in the transcripts of pro-inflammatory cytokines (IL6 and IL1β) and in an acute-phase protein (SERPINA3) in the schizophrenia midbrain. These changes were all of a greater magnitude than the increases previously reported in the cerebral cortex [4–6]. Distinct from our previous studies in cortex [4–6], TNFα and IL6ST mRNAs were also increased in the midbrain, suggesting more extensive immune changes in this subcortical brain area as compared to the cortex.

Our stratification strategy classified ~ 46% of people with schizophrenia as having elevated cytokine-associated immune profiles in the midbrain, whereas ~ 54% of schizophrenia cases have cytokine levels comparable to control subjects. Hence, our study defines distinct immune biotypes in schizophrenia based on the transcriptional profiling of a disease-relevant brain area that is known to contain dysfunctional dopamine cells [24, 25]. Our findings further suggest that diagnostic group differences in immune-related gene expression are accounted for, or even driven by, a substantial subgroup of schizophrenia cases characterized by a high cytokine status. In addition, the increased immune-related gene expression is not limited to transcripts used to generate the clusters and thus the stratification may be more broadly informative of an activated immune/cytokine state within the midbrain. The stratification of schizophrenia cases into high and low immune biotypes in the midbrain is also consistent with the percentage of biotype-specific changes found in the dorsolateral prefrontal cortex [4, 6], orbitofrontal cortex [5], and periphery [16, 17]. It remains to be determined, however, whether individuals with a high cytokine status in midbrain concomitantly show a high cytokine status in other brain areas such as cortex and/or in peripheral tissue such as blood.

Our study also identified strong positive correlations between daily and/or lifetime CPZ dose and some immune markers (i.e., three cytokines and microglial markers, but not an astrocyte marker) in the midbrain of people with schizophrenia, supporting the notion that antipsychotic treatment influences immune profiles in the brain [8, 56, 58]. However, it remains unknown whether this correlative relationship indicates that increased immune markers are caused by antipsychotics (supported by [15]), or alternatively, whether these patients were more symptomatic and thus required higher antipsychotic doses to attenuate symptoms. Indeed, first-episode schizophrenia patients with high circulating cytokines prior to treatment do worse with respect to their long-term symptom outcomes [20], and a recent preclinical study in rats showed that antipsychotics alone (but dependent on the dose and duration) are sufficient for microglia activation [58]. However, the meta-analysis of Goldsmith et al. [57] suggests that high circulating pro-inflammatory cytokines in first-episode patients and in chronic patients experiencing an acute psychotic episode are reduced by treatment. This is consistent with our observation that risperidone decreases IL1β mRNA in cultured endothelial cells. Additional lines of evidence further indicate that at least some of the immune-related changes in the midbrain of people with schizophrenia may emerge independently of antipsychotic exposure. First, it was previously demonstrated that chronic treatment with olanzapine or haloperidol did not change IL1β or IL6 mRNA levels in macaque cortex, although this study is limited by small sample sizes [7]. However, this is in line with our study showing that neither haloperidol nor clozapine changed IL1β or IL6 gene expression in cultured brain endothelial cells, which are a major source of these cytokines [59]. Second, consistent with our results from human midbrain, particularly SERPINA3 that did not correlate with antipsychotic exposure (nor was SERPINA3 expression increased by antipsychotics in cultured endothelial cells), we found increased expression of several immune markers in the midbrain of adult MIA offspring relative to controls. As MIA offspring and controls did not receive any antipsychotic medication, we can exclude a possible confound arising from drug exposure in this model. Moreover, the extent to which MIA offspring can be stratified into high (~ 41%) and low inflammatory (~ 59%) subgroups is markedly similar to the stratification of human cases into high and low inflammatory biotypes. Although we cannot rule out an effect of antipsychotics on cytokine gene expression in the postmortem human study, translating the findings from the MIA model to the observations in the postmortem human tissue would suggest that early (infection-mediated) neurodevelopmental abnormalities might be one of the etiological factors contributing to persistent inflammation-related immune changes in the midbrain of people with schizophrenia.

If we consider that (chronically) elevated inflammatory processes are detrimental to the brain, our data indicate that midbrain inflammation may be a contributing factor for presynaptic dopamine abnormalities relevant to schizophrenia. In support of this hypothesis, we and others previously showed that adult MIA offspring display a number of dopaminergic alterations at the level of the midbrain and its main innervation areas, including nucleus accumbens, caudate putamen, and medial prefrontal cortex [34–40]. These presynaptic abnormalities include increased and decreased tyrosine hydroxylase expression in the midbrain and medial prefrontal cortex [39, 46], respectively, reduced spontaneous firing rate, and population activity of ventral tegmental dopamine neurons [40], increased tyrosine hydroxylase expression in the nucleus accumbens [39], and elevated basal dopamine release in nucleus accumbens, caudate putamen, and globus pallidus [60–62]. Interestingly, acute exposure to pro-inflammatory factors such as IL1β and IL6 has similarly been shown to stimulate tyrosine hydroxylase expression and dopaminergic activity in vivo and in vitro [27, 29, 63]. In view of these findings, it is likely that increased expression of immune-related markers in the midbrain may contribute to some of the presynaptic dopaminergic effects induced by MIA, [39, 40, 46, 60–62] and possibly to those existing in the midbrain of people with schizophrenia as well [24, 25].

As microglia and astrocytes are two major sources of cytokine and chemokine expression in the brain parenchyma [31, 32], we expected the elevation of inflammatory markers in the midbrain of schizophrenia cases and MIA offspring to be associated with microglial and astrocytic anomalies. This expectation was, however, fulfilled only partially. When analyzed based on diagnosis, microglial cell number and gene expression of multiple microglial markers were unchanged in the human midbrain. However, increased AIF1, CD68, and TSPO mRNA was identified in high immune schizophrenia cases, although the number of microglia per se seemed unchanged. This suggests that qPCR may be more sensitive to microglia changes and that the increase in microglia markers depends on immune subgroup. Neither AIF1 and TSPO mRNA nor microglia density and morphology were altered by MIA at the group level, whereas increased AIF1 and TSPO mRNA was identified in a subgroup of MIA-exposed offspring that were characterized by a high immune profile. The astrocyte marker, GFAP mRNA, was increased based on diagnosis (human cohort) but, along with GFAP cell density, not after prenatal immune activation (mouse cohort). Intriguingly, however, in both cohorts GFAP mRNA was increased in the high immune subgroups. In as much as increased AIF1 and TSPO transcription can be assumed to index microglial activation, and GFAP transcription to index astrocyte activation [33], our findings thus indicate that potentially only a subgroup of schizophrenia patients or poly(I:C)-exposed offspring are prone to increased microglia-related and astrocyte-related immune activity.

We did not identify any microglia morphological changes in the mouse midbrain. However, alterations in microglia activation can occur in the absence of morphological changes as reported in a study showing increased numbers of microglia in the brain expressing CD68, a lysosomal protein associated with phagocytosis, without concomitant changes in morphology following exposure of mice to chronic social defeat [64]. In view of the multifaceted and highly dynamic nature of microglia and astrocytes [31, 65], it also remains elusive whether changes in the activation status of these cells are consistent throughout an individual’s illness, or whether this is a dynamic process, with individuals moving in and out of the high immune classification. This question can potentially be addressed in future studies using longitudinal investigations of central (and peripheral) inflammation in the MIA model.

Our study has several limitations. Although the extent to which schizophrenia cases and MIA offspring can be stratified according to low and high immune status in the midbrain is remarkable, we do not know the underlying mechanisms responsible for this stratification. Elucidating these mechanisms seems warranted because distinct immune-related biotypes may be meaningful with respect to shaping differential vulnerability for neuronal and behavioral abnormalities [66]. Also, interpreting changes on immune state based on mRNA alone is a limitation of the human studies and future studies should also determine whether protein levels and immune activity levels change in the midbrain. In the mouse MIA model, however, we found a remarkable correspondence between cytokine changes at the mRNA and protein level, suggesting that even relatively subtle alterations in cytokine gene expression, such as those present in MIA-exposed offspring, translate into corresponding changes at the protein level. Another limitation of our study is that the possible influence of sex was not thoroughly examined in the human cohort, owing to loss of statistical power when the groups were split by sex. Assessing the influence of sex would be desirable in view of the findings derived from our study, which revealed some sex-dependent effects of MIA on midbrain inflammatory markers and the study of Hui et al. [67], which identified sex-specific changes in the MIA model in cortex, cerebellum, and hippocampus. In an early gestational (GD 9) MIA model, Hui et al. found larger increases in IL1β gene expression in the cortex of MIA-exposed males compared to MIA-exposed female offspring. Using a late gestational (GD17) MIA model, our study identified larger increases in the expression of TNFα and IL1β mRNAs in the midbrain of MIA-exposed females compared with males. Our data thus indicate that female brains may be more severely impacted than male brains in response to late prenatal immune activation, at least when considering immune-related changes in the midbrain. Our study did not determine the cellular sources of altered cytokine and acute-phase protein transcription. Besides microglia and astrocytes, other parenchymal cell types may contribute to these effects, including neurons [31, 65, 68, 69]. Finally, postmortem schizophrenia research has inherent limitations. The full impact of antemortem effects on postmortem brain cannot be ruled out, nor can the possible confounds of antipsychotic treatment in schizophrenia cases be excluded. However, although it is possible that events surrounding/contributing to the death, or occurring in the PMI and/or treatment with antipsychotics, may modify the immune markers measured in our human work, antemortem factors and antipsychotics do not contribute to the equivalent changes in immune markers measured in the mouse midbrain.

In conclusion, our findings implicate inflammation-related immune abnormalities in the midbrain of a subgroup of people with schizophrenia. Prenatal exposure to immune activation may be a relevant etiological factor, contributing to a high immune status in this subgroup. Our findings encourage future attempts to determine whether a high immune status correlates with, or even causes, more-pronounced behavioral changes and cognitive deficits. In addition, studies of astrocyte morphology and spatial localization of microglia cells (e.g., proximity to dopamine neurons) [67] are needed to better understand the potential contribution of microglia and astrocytes in these associations. Future studies are also crucial to determine whether molecular measurements of the dopaminergic system, which are dysregulated in the midbrain in schizophrenia [25], differ according to inflammatory status. Also, preclinical studies are required to determine how antipsychotics may modulate gene expression of immune markers, particularly of the cytokines used to generate the low and high immune groups.

## Supplementary information

Supplementary Material - Final

Supplementary Figure Legends - Final

Supplementary Figure 1

Supplementary Figure 2

Supplementary Figure 3

Supplementary Figure 4

Supplementary Figure 5
