# Supplementary material for: Increased levels of midbrain immune-related transcripts in schizophrenia and in murine offspring after maternal immune activation
Source: Mol Psychiatry. 2019 Jun 5;26(3):849–63. doi: 10.1038/s41380-019-0434-0 (PMC7910216; doi:10.1038/s41380-019-0434-0)
Supplement: Supplementary file 1 — Supplementary Material - Final [file 41380_2019_434_MOESM1_ESM.docx]

**SUPPLEMENTARY INFORMATION**

**Increased levels of midbrain immune-related transcripts in schizophrenia and in murine offspring after maternal immune activation**

Tertia D. Purves-Tyson PhD^1,2^ Ulrike Weber-Stadlbauer PhD^3^, Juliet Richetto PhD^3^, Debora A Rothmond MSc^1^, Marie A. Labouesse PhD^4,5^, Marcello Polesel MSc^6^, Kate Robinson BSc(Hons)^1^, Cynthia Shannon Weickert PhD^$1,2^ and Urs Meyer PhD^$3,7^

^1^Schizophrenia Research Laboratory, Neuroscience Research Australia, Sydney, NSW 2031, Australia

^2^School of Psychiatry, Faculty of Medicine, University of New South Wales, Sydney, NSW 2052, Australia

^3^Institute of Pharmacology and Toxicology, University of Zurich-Vetsuisse, Zurich, Switzerland.

^4^Physiology and Behavior Laboratory, ETH Zurich, Schwerzenbach, Switzerland.

^5^Department of Psychiatry, College of Physicians and Surgeons, Columbia University, 1051 Riverside Drive, NYC 10032, NY, USA.

^6^Institute of Anatomy, University of Zurich, Zurich, Switzerland.

^7^Neuroscience Centre Zurich, University of Zurich and ETH Zurich, Switzerland.

*^$^ Joint Corresponding Authors:*

*Cynthia Shannon Weickert, Ph.D.*

Neuroscience Research Australia

Schizophrenia Research Laboratory

139 Barker Street, Margarete Ainsworth Building, Level 5

Randwick NSW 2031 Australia

T: +61 2 9399 1000

F: +61 2 9399 1005

E: cyndi@neura.edu.au

*Urs Meyer, Ph.D.*

Institute of Pharmacology and Toxicology

University of Zurich - Vetsuisse

Winterthurerstrasse 260

CH-8057 Zurich

T: +41 44 635 88 44

F: +41 44 635 89 10

E: [urs.meyer@vetpharm.uzh.ch](mailto:urs.meyer@vetpharm.uzh.ch)

**Supplementary Methods & Materials**

***Human postmortem cohort additional information***

Details of the midbrain postmortem cohort have been previously published^1^.

**Cause of death/agonal state**

Agonal state, on a scale of 1-4, is based on manner and/or cause of death, considering the type of death and the length of the terminal phase.^2^ All postmortem cases in this entire cohort were classified in the agonal state categories of 1 or 2. Most control cases died of cardiac failure (22) not associated with long-term hospitalization, 4 died of cardiac failure associated with respiratory problems, 1 from trauma and 1 from toxicity, 1 unrecorded in notes. No control cases died by suicide, however, 7 schizophrenia cases were suicide completers. Other causes of death in schizophrenia patients were cardiac failure (10), respiratory problems (6), cancer (1), renal failure (1), peritonitis (1) and unrecorded in notes (2)

**Additional assessment of antipsychotic treatments and symptoms**

Clozapine use is generally only recommended after at least 2 trials of other antipsychotics have failed^3^, thus clozapine treatment at time of death (n=7) was used as an indicator of possible treatment resistance versus all other antipsychotics at time of death (n=21) (total=28). Antipsychotic treatment was also broken down into those that received mostly first generation antipsychotics (n=17) and those treated with mostly second generation antipsychotics (n=11). The schizophrenia cases were also diagnosed with either more prevalent positive symptoms (n = 22) or more prevalent negative symptoms (n = 7) with one unknown.

***Taqman gene expression assays for quantitative real-time PCR***

Information regarding Taqman gene expression assays is provided in Supplementary Table 1.

***Quantification of microglia by immunohistochemistry in human midbrain***

Frozen 14μm human midbrain sections were fixed in 4% paraformaldehyde and treated to inhibit endogenous peroxidase activity. Slides were blocked in 10% normal horse serum (Vector Laboratories, Burlingame, CA) and incubated (4°C overnight) with 1:1000 HLA-DR primary antibody (M0775; Dako, North Sydney, NSW, Australia). Tissue was washed with phosphate buffered saline (PBS) and incubated in 1:1000 horse anti-mouse biotinylated secondary antibody (BA-2000; Vector Laboratories) before conjugation with avidin-peroxidase complex (VectaStain kit PK-4000, Vector Laboratories). Antibody binding was visualized with DAB (Sigma-Aldrich, Castle Hill, NSW, Australia), washed, dehydrated, stained with Nissl and coverslipped with permount. Density of HLA-DR positive (HLA+) cells was examined in two dimensions with a 20X objective (Nikon Eclipse 80i light microscope, Coherent Scientific, Hilton, SA, Australia). Stereo-Investigator (v23, MicroBrightField, Williston, VT) was used to randomly place a grid containing twelve 140x140µm boxes in two distinct regions within the substantia nigra, identified by the presence of neuromelanin containing cells ventral to the red nucleus and referencing tyrosine hydroxylase stained slides from the same case. Two images were taken per slide within the substantia nigra (20X objective) and HLA+ cells counted in six random boxes of the 140x140µm grid, with two permissive and two non-permissive edges, in each image (total area counted was 0.2353mm^2^). Counting was performed by two researchers (blind to diagnosis) and cell density expressed as cell/mm^2^. The data sets were positively correlated (r=0.69, n=53, *p*<0.0001) and average data is presented.

***Maternal immune activation (MIA) model***

Female and male C57BL6/N mice were obtained from Charles River Laboratories (Sulzfeld, Germany) and kept in-house for minimum of two weeks before breeding. Upon arrival, they were kept in conventional open cage systems, which were made of transparent polysulfone cage bodies (Tecniplast, Buguggiate, Italy), with cage dimensions of 140 mm (high; without outer cage lid) × 160 mm (wide) × 320 mm (long) as fully described elsewhere^4^. Animal rooms were temperature and humidity controlled (21±1°C, 55±5%) and under a 12 h reversed light–dark cycle. Mice had *ad libitum* access to food (Kliba 3436, Kaiseraugst, Switzerland) and water. All procedures were approved by the Cantonal Veterinarian’s Office of Zurich (approval nr. ZH 172/2015). All efforts were made to minimize the number of animals used and their suffering.

For the MIA model, female mice were subjected to a timed mating procedure as described previously.^5^ Pregnant dams on gestation day (GD) 17 were subjected to either a single injection of poly(I:C) (potassium salt; Sigma–Aldrich, Buchs, St. Gallen, Switzerland) or vehicle solution. Poly(I:C) (5 mg/kg; calculated based on the pure form of poly(I:C)) was dissolved in sterile pyrogen-free 0.9% NaCl (vehicle) solution to yield a final concentration of 1 mg/ml and was administered intravenously (i.v.) into the tail vein under mild physical constraint as described previously.^5^ The dose of poly(I:C) was selected based on our previous dose-response studies.^5^ We also verified previously that this poly(I:C) administration regimen is effective in terms of eliciting cytokine-associated inflammatory response in maternal and fetal tissues.^6,7^

The selected gestational window (i.e. GD 17) in the mouse roughly corresponds to human gestational weeks 13 to 14 in terms of midbrain development (http://translatingtime.org/translate). It was selected based on (1) our previous immunohistochemical, imaging and behavioral studies showing dopamine-related cellular and volumetric changes in the ventral midbrain and increased amphetamine sensitivity in adult offspring exposed to MIA at this gestational time point^8-10^ and (2) human epidemiological studies suggesting that MIA during late-first and early-second trimester of human pregnancy are associated with dopamine-related psychotic disorders in the offspring.^11,12^

In total, 14 pregnant dams were injected with vehicle solution, whereas 13 dams received poly(I:C) treatment. The resulting offspring were weaned on postnatal day (PND 21) and kept until adulthood (PND 120) as described below. To avoid possible litter effects and confounds arising from technical replicates,^13^ only 1-2 male and female offspring were randomly selected from each litter, resulting in a group size of *n* = 32 (16 males, 16 females) per treatment group for the transcriptomic analyses and *n* = 10 (5 males, 5 females) for the immunohistochemical analyses (see below). An additional 12 dams (6 in each treatment group) were used to generate offspring to be assigned to cytokine protein measurement in the ventral midbrain (see below). One male and female offspring were randomly selected from each litter, resulting in a group size of *n* = 12 (6 males, 6 females) per treatment group for cytokine protein measurements. Estimations of sample size were based on previous studies from our laboratory.^1-6^ Both males and females were included in order to identify possible sex-dependent effects of MIA.

All offspring were weaned and sexed on postnatal day (PND) 21. Littermates of the same sex were caged separately and maintained in groups of 3 to 4 animals per cage. They were kept in conventional open cage systems, which were located in temperature- and humidity-controlled holding rooms under a reversed light–dark cycle and *ad libitum* food access as described above. Offspring from the first cohort of dams (see above) were randomly assigned to either the transcriptomic or immunohistochemical analyses when they reached adulthood (i.e., on PND 120), whereas offspring from the second cohort of dams (see above) were randomly assigned to protein cytokine measurements at the same age (i.e., on PND 120).

***Mouse brain collection for transcriptomic and cytokine protein analyses***

Mice were killed by decapitation for the purpose of the transcriptomic analyses in the MIA model. The brains were rapidly extracted from the skull (within < 30 s), immediately frozen on powdered dry ice and kept at -80 °C until further processing. Frozen coronal sections were then prepared using razorblade cuts along the following coordinates with respect to bregma: -2.9 to -3.8 mm. Micropunches of the substantia nigra (comprising pars compacta and reticulata subregions) were then generated using a micropunch needle (1 mm in diameter) as described before.^9^ The punches were then immediately processed further for RNA extraction (see *main text*).

***Mouse brain collection for immunohistochemical analyses***

The animals were deeply anesthetized with an overdose of Nembutal (Abbott Laboratories, North Chicago, IL, USA) and perfused intracardially with 0.9% NaCl, followed by 4% phosphate-buffered paraformaldehyde (PFA) solution containing 15% picric acid. The dissected brains were postfixed in the same fixative for 6 h and processed for antigen retrieval involving overnight incubation in citric acid buffer (pH 4.5) followed by a 90 s microwave treatment at 480 W according to protocols established before.^7^ The brains were then cryoprotected using 30% sucrose in PBS, frozen with powdered dry ice, and stored at −80°C until further processing. Perfused brain samples were cut coronally at 30 μm thickness from frozen blocks with a sliding microtome (HM430, Histocom AG, Zug CH). Eight serial sections were prepared for each animal and, after rinsing in PBS, stored at −20°C in antifreeze solution (30% glycerol and 30% ethylene glycol in PBS at 25 mM and pH 7.4) until further processing.

***Mouse immunohistochemistry***

For immunohistochemical staining, the slices were rinsed three times for 10 min in PBS and blocked in PBS containing 0.3 % Triton X-100 and 10 % normal serum for 1 h at room temperature. The following primary antibodies were used: rabbit anti-AIF (=Iba1; Wako, Neuss, Germany; cat# 019-19741; 1:3,000 dilution), rat anti-CD68 (AbD Serotec, Oxford, UK; cat# MCA1957GA; 1:3,000 dilution), and mouse monoclonal anti-GFAP (EMD Millipore, Billerica, USA; cat# MAB360; 1:5,000 dilution). The primary antibodies were validated previously.^7,14^ They were diluted in PBS containing 0.3 % Triton X-100 and 2 % normal serum, and the sections were incubated free-floating overnight at room temperature. After three washes with PBS (10 min each), the sections were incubated for 1 hr with the biotinylated secondary antibodies diluted 1:500 in PBS containing 2 % NGS and 0.3 % Triton X-100. Sections were washed again three times for 10 min in PBS and were then incubated with Vectastain kit (Vector Laboratories, Burlingame, CA, USA) diluted in PBS for 1 h. After three rinses in 0.1 M Tris-HCl, pH 7.4, the sections were stained with 1.25 % 3,3-diaminobenzidine and 0.08 % H_2_O_2_ for 10–15 min, rinsed again four times in PBS, mounted, dehydrated, and coverslipped with Eukitt (Kindler, Freiburg, Germany).

***Mouse stereology***

The numbers of AIF-, CD68-, or GFAP-immunoreactive cells were determined by unbiased stereological estimations using the optical fractionator method.^15^ With the aid of the image analysis computer software Stereo Investigator (version 6.50.1; MicroBrightField, Williston, VT, USA), every section of a one-in-eight series was measured, resulting in an average of 4 to 5 sections per brain sample. The following sampling parameters were used: (1) a fixed counting frame with a width of 40 μm and a length of 40 μm; and (2) a sampling grid size of 130 × 80 μm. The counting frames were placed randomly at the intersections of the grid within the outlined structure of interest (substantia nigra, encompassing pars compacta and pars reticulata from Bregma -2.80 to -3.64mm) by the Stereo-Investigator (v6.50.1; MicroBrightField) software. The cells were counted by an experimenter blinded to treatment. Counting was performed following the unbiased sampling rule using the 40× oil lens [numerical aperture (NA), 1.3] and included in the measurement when the immunoreactive cells came into focus within the optical dissector.^16^ For the stereology, the mean ± s.e.m of Gundersen coefficient of error (CE) of AIF+ cells was 0.097±0.004 for poly(I:C)-exposed offspring and 0.094±0.003 for vehicle-exposed offspring; the mean ± s.e.m of Gundersen CE for CD68+ cells was 0.103±0.006 for poly(I:C)-exposed offspring and 0.099±0.005 for vehicle-exposed offspring; and the mean ± s.e.m of Gundersen CE for GFAP+ cells was 0.112±0.010 for poly(I:C)-exposed offspring and 1.102±0.006 for vehicle-exposed offspring. AIF+, CD68+ and GFAP+ cells were expressed as cells/mm^3^.

***Assessment of microglia morphology in mice***

The assessment of microglia morphology was conducted according to previous procedures.^7^ In brief, AIF-immunoreactive microglia were visualized under the 63 x oil lens [numerical aperture (NA), 1.4] using a Zeiss Axiophot microscope. Various parameters of microglia cell morphology, including cell soma size, number of primary processes, and number of branch points per process, were assessed in SN of poly(I:C)-exposed and control mice. A counting frame of 100 μm × 100 μm was randomly placed into three sections of a one-in-eight series. All microglia cells captured by the counting frame were included in the morphological analyses, expect when microglial processes were obscured by either background labeling or other cells. Three to four microglia cells per section were traced using the software Stereo Investigator (version 6.50.1; MicroBrightField), for which cell soma size, number of primary processes, and number of branch points per process were estimated, giving a total of 9 to 12 cells per region of interest.

***Mouse cytokine protein measurements***

Micropunches of the substantia nigra (comprising pars compacta and reticulata subregions; see above) were used to generate homogenates for cytokine protein measurements as described elsewhere^7,14^. In brief, the micropunches were homogenized (TissueTearor; BioSpec Products, Bartlesville, OK, USA) for 10 s in 300-μl lysis buffer (50 mM Tris-HCl (pH 7.4), 0.6 M NaCl, 0.2 % Triton X-100, 0.5% bovine serum albumin, and protease inhibitors [1 mM benzamidine, 0.1 mM benzethonium chloride, and 0.1 mM phenylmethylsulfonyl fluoride]), sonicated (Vibra Cell; Sonics & Materials, Newtown, CT, USA) for 20 s at 10 mV, and centrifuged. The midbrain supernatants were aliquoted and stored at –80 °C until required.

Cytokine proteins in midbrain homogenates were quantified using a customized Meso-Scale Discovery (MSD) V-Plex electrochemiluminescence assay for mice, which allows ultralow detection of multiple cytokines in mouse supernatants^7,14^. Based on transcriptomic analyses, V-plex plus 96-well plates were coated with primary antibodies directed against interleukin IL1β, IL6, TNFα and treated with the detecting antibodies pre-labeled with SULFO-TAG™ (MSD, Rockville, Maryland, USA). The plates were read using the MESO SECTOR S 600 (MSD) imager and analyzed using MSD’s Discovery Workbench analyzer and software. All assays were run in triplicate. The detection limits were 0.08 pg/ml for IL1β, 0.20 pg/ml for IL6, and 0.04 pg/ml for TNFα. To express midbrain cytokine levels, the cytokine concentrations quantified in the midbrain lysates were normalized to the amount of total protein present in the corresponding lysates.

***Primary statistical analyses***

In the human cohort, independent sample two-tailed *t*-tests were performed to compare demographic variables, housekeeper gene expression and the geomean of the three housekeepers (geomean3), between the diagnostic groups. Grubb’s test was used to identify diagnostic/treatment group outliers (0-2 individuals/diagnostic group/gene of interest). Correlations between demographic variables (age at death, pH, PMI, RIN) housekeeper genes and geomean3 were previously reported [Supplementary Material^1^]. Briefly, age at death and PMI did not correlate with the expression of the housekeeper genes or with geomean3 (all r<-0.11; p>0.5; n=57), whereas brain pH and RIN were positively correlated with geomean3 and gene expression of all three housekeepers (all r>0.39; p<0.05). Genes of interest were normalized to geomean3 to account for the impact of variations in pH or RIN. Normalized gene expression levels were tested for normality (Shapiro-Wilk test). Human IL1β, IL6, SERPINA3, TNFα,IL8, CD68, GFAP, TSPO, IL1A and IL17RA mRNAs were log transformed and AIF1 mRNA was square root transformed to achieve normality. Pearson’s or Spearman’s (duration of illness and age at death) correlations were used to test demographic variables (pH, RIN, PMI) and normalized transcripts of interest. No transcript correlated with duration of illness or age at death, whereas, some correlated with pH only (AIF1, SERPINA3, TNFα, IL6, IL8), RIN only (IL18), or both PH and RIN (IL6ST, GFAP), or with neither (IL1β, CD68, TSPO, IL1A, IL17RA). Analysis of variances with covariates (ANCOVAs) were used to detect differences in mRNA or HLA density (age at death) between diagnostic groups with covariates or Student’s *t*-test if there were no covariates. The effect of antipsychotics on mRNA was explored with Spearman’s correlations^1^. Mouse qPCR, cytokine protein data, microglial cell density and astrocyte density were analyzed by two-way (prenatal treatment x sex) analysis of variance (ANOVA) followed by Fisher’s least significant difference (LSD). Sex was not used as a grouping factor in the human cohort due to insufficient statistical power for females. Cell culture experiments were analysed by one-way ANOVAs, followed by LSD.

High/low immune subgroups were defined using two-step cluster analyses^17,18^ on the entire case/control human postmortem cohort as previously reported^19^ and on the entire poly(I:C)-exposed/control offspring cohort. Missing values were replaced using an expectation maximization algorithm (SPSS). In humans, the overall model quality was >0.5, with predictors of least importance removed until all predictors contributed significantly to the model (predictor importance >0.4 on a scale of 0-1.0). The robustness of the human clusters was by resampling of the data and running the cluster algorithm 20 times on 54 cases (n – 3), randomly selected without replacement. The final mRNAs used to define the immune subgroups were SERPINA3, IL6, IL1β and TNFα. In mice, SERPINA3, IL6, IL1β, IL18, TNFα mRNAs were used to generate immune subgroups, with a model quality >0.4.

The human and mouse cohorts were split into immune and diagnosis/treatment subgroups. In the human cohort, there were three immune/diagnosis subgroups (control/low immune, schizophrenia/low immune, schizophrenia/high immune). In the mouse cohort, four treatment/immune groups were identified (vehicle/low immune, vehicle/high immune, poly(I:C)/low immune, poly(I:C)/high immune). Three mice were vehicle/high immune and were omitted from analysis (shown on graphs for visual comparison). Chi squared tests were used to determine differences in categorical variables (e.g. sex, agonal state, smoking) within the immune and diagnosis/treatment subgroups. In the human cohort, ANCOVAs (covariates as above) were used to determine differences in mRNAs and HLA+ density between the inflammatory/diagnosis subgroups by pairwise comparisons. In the mouse cohort, the three immune/treatment groups were compared by ANOVA followed by pairwise comparisons, or by Mann-Whitney tests.

***Secondary statistical analyses: Antipsychotic treatment assessment and impact of mode of death and smoking on gene expression***

The effect of antipsychotics (lifetime, mean daily and last dose chlorpromazine equivalents) on gene expression was explored with Spearman’s correlations. Comparisons were made between schizophrenia cases divided into those treated with mostly first generation antipsychotics and those treated with mostly second generation antipsychotics and between cases using clozapine at time of death (possible treatment resistance) versus those on antipsychotics other than clozapine at time of death. Additionally, the effect of mostly positive or mostly negative symptoms, suicide status and presence of depression symptoms during lifetime or no depression symptoms were explored in the schizophrenia cases. Student’s two-tailed *t*-tests (equal variances not assumed) were used to explore differences in gene expression in the schizophrenia group based on antipsychotic treatment, symptoms, depression and suicide. Student’s two-tailed *t*-tests (equal variances not assumed) were also used to explore differences in gene expression in the schizophrenia and control groups, separately, based on mode of death, and smoking at time of death. These analyses are exploratory as the group sizes are modest.

**Supplementary Results**

***Effect of sex on gene expression in MIA offspring***

SERPINA3 mRNA increased 13% in midbrain from male compared to female mice (sex effect: F=5.490, df=60,1, *p*=0.022, Supplementary Fig. 1a) and there was no treatment × sex effect (F=0.019, df=60,1, *p*=0.89). There was a trend for TNFα mRNA to be changed according to sex (F=3.201, df=59,1, p=0.079, Supplementary Fig. 1b) and a trend toward a sex × treatment interaction (F=2.898, df=59,1, p=0.094)

IL6 mRNA increased 16% in male offspring (sex effect: F=8.172, df=1,60, p=0.006, Supplementary Fig. 1c) and, although IL18 mRNA differed according to sex (F=6.757, df=1,56, p=0.012, Supplementary Fig. 1d), neither IL6 nor IL18 mRNAs showed a sex × treatment interaction (both F<2.196, df=60/56,1, p>0.05).

IL1β mRNA (Supplementary Fig. 1e) was not changed according to sex (F=0.190, df=60,1, p=0.665), but showed a treatment × sex interaction (F=6.093, df=1,60, p=0.016) with IL1β mRNA only increased (46%) in the midbrain of female poly(I:C)-exposed offspring.

***Altered cytokine proteins in the midbrain of MIA offspring***

MIA-exposed offspring displayed a significant increase in midbrain TNFα (main effect of prenatal treatment: F=12.72, df=20,1, *p*=0.002) and IL1β (main effect of prenatal treatment: F=5.56, df=20,1, *p*=0.029) protein levels, whereas midbrain IL6 protein levels were not affected by MIA (F<0.04) (Supplementary Fig. 2a,b,c). For all cytokine protein measurements, there was no main effect of sex (all F<1.5) or interaction between sex and prenatal treatment (all F<0.5), suggesting that MIA affected midbrain cytokine proteins similarly in male and female offspring (Supplementary Fig. 2d,e,f).

***No effect of sex on markers of microglia or astrocytes in MIA offspring***

In the mouse cohort, AIF, TSPO and GFAP mRNA were not modified by sex, and there was no treatment × sex interaction (all, F<0.954, df=1,51-60, *p*>0.05; Supplementary Fig. 3a,b,c). Likewise, AIF+ (Supplementary Fig. 3d), CD68+ (Supplementary Fig. 3e) microglial and GFAP+ astrocytic (Supplementary Fig. 3f) cell densities showed no differences between male and female mouse offspring, and there was no treatment × sex interaction (all F<1.0, df=1,60, *p*>0.05). There was no effect of sex or treatment × sex interactions on AIF+ microglial cell soma size, number of primary processes or number of branch points per process (all F<1.1, df=1,16, *p*>0.05) (data not shown).

***Transcripts used to generate the high and low immune groups in the midbrain in schizophrenia and the MIA model are elevated in a subgroup of schizophrenia cases and MIA offspring***

As expected, SERPINA3 and TNFα (Supplementary Figure 4a,b) mRNAs (both F>12.31, df=1,52, *p*<0.0001) were increased 484% and 200% in the schizophrenia/high compared to the control/low immune subgroup, and 531% and 200% compared to the schizophrenia/low immune subgroup, respectively (all *p*<0.0001). IL6 and IL1β (Supplementary Figure 4c,d) mRNAs (F>22.89, df=2,51/52, *p*<0.0001) in the schizophrenia/high immune subgroup were elevated above both the control/low (1210% and 499%) and the schizophrenia/low immune subgroups (1176% and 507%) (all *p*<0.0001). IL8 and IL18 mRNAs were not significantly different between the subgroups (F<1.625, df=53/54, *p*>0.05, Supplementary Figure 4e,f), whereas IL6ST mRNA (F=6.05, df=2,53, *p*=0.004, Supplementary Figure 4g) was increased 20% in both the schizophrenia/high and schizophrenia/low immune subgroups compared to the control/low immune subgroup (both *p*<0.05).

Also as expected, in the MIA model, there was a significant effect of immune subgroup on the transcript levels of SERPINA3 (F>4.247, df=2,58, *p*<0.05), TNFα (*U*=20.574, N=61, *p*<0.0001), IL6 (F=5.982, df=2,57, *p*=0.004) and IL1β (*U*=21.748, *N*=61, *p*<0.0001) (Supplementary Figure 4h-k). SERPINA3, TNFα, IL6, and IL1β mRNAs were all increased in the poly(I:C)/high immune subgroup compared to the vehicle/low immune and the poly(I:C)/low immune subgroups (all between 11-66%, *p*<0.05). In contrast, although a similar pattern of expression was seen between immune subgroups, there was no significant effect on IL18 mRNA in the midbrain (*U*=4.074, *N*=59, *p*=0.13; Supplementary Figure 4l) in the MIA model.

***Effect of antipsychotics and clinical state (depression, suicide) on gene expression***

The effect of antipsychotics (lifetime, mean daily and last dose chlorpromazine equivalents) on gene expression was explored with Spearman’s correlations. TNFα, IL1β, IL6, IL1A, IL17RA, AIF, and CD68 mRNAs were positively correlated with daily and TNFα, IL6, IL17RA and CD68 mRNAs with lifetime exposure (all r>0.5, *p*<0.05). SERPINA3, IL8, IL18, IL6ST, TSPO and GFAP mRNAs did not correlate with any chlorpromazine equivalents (all *p*>0.05), and no mRNA correlated with duration of illness (Table 2, main manuscript).

No mRNA showed a difference with clozapine at time of death (n=7, possible treatment-resistant schizophrenia) compared to those treated with other antipsychotics (n=21) (all; *t*<1.60, df=7.01-14.60, *p*>0.05). IL6 and IL8 mRNAs were increased in the midbrain of schizophrenia cases that received mostly first generation (n=17) antipsychotics compared to schizophrenia cases that received mostly second generation (n=11) antipsychotics (both; *t*>2.27, df=15.54-23.01, *p*<0.05). When examining symptoms, there were no changes in immune-related gene expression when schizophrenia cases displaying mostly positive (n=18) or negative (n=7) symptoms were compared (all t<0.645, df=7.62-16.10, *p*>0.05). In addition, although only exploratory due to low group sizes, there were no differences in the distribution of mostly first generation/mostly second generation antipsychotics, treatment resistant/responsive or mostly positive/mostly negative symptoms, between the low and high cytokine groups in the schizophrenia cases (all χ^2^ < 2.047, *p* > 0.05).

No gene of interest varied significantly in the schizophrenia group based on the presence of depression symptoms within their lifetime (depression symptoms/no depression symptoms n=8/18; all t<1.13, df=14.07-23.17, *p*>0.05) or whether or not they had died by suicide (suicide negative/suicide positive n=20/7; all t<0.645, df=7.62-16.10, *p*>0.05). Suicide is associated with increased microglia density in postmortem brain in schizophrenia^20^ and increased cytokine mRNAs in postmortem brain in suicide completers^21^. We are unable to assess this in our study due to low number of suicide completers, but note that in our cohort there are more suicide completers in the low immune group (6/15) than in the high immune group (1/13).

***Impact of mode of death and smoking on gene expression***

In the control cases there were 18 “rapid” and 10 “intermediate” mode of death classifications, whereas in the schizophrenia cases there were 16 “rapid” and 9 “intermediate” mode of death classifications. TNFα mRNA was increased in the “rapid” mode of death group compared to an “intermediate” mode of death group in both the control and schizophrenia groups (both *t*>2.68, df=18.83-19.70, *p*<0.05). AIF mRNA increased in the rapid mode of death group compared to the intermediate group in the schizophrenia cases (*t*=2.94, df=18.39, *p*=0.009) but not in the control cases (*t*=0.922, df=17.52, *p*=0.37). No other genes of interest were changed in either the control or schizophrenia cases according to their “mode of death” classification (all t<2.68, df=14.01-24.81, *p*>0.05).

Smoking status at time of death was known in a subset of post-mortem cases. In the control cases there were 11 smokers and 12 non-smokers, whereas in the schizophrenia cases there were 16 smokers and 6 non-smokers. SERPINA3, the only mRNA modified by smoking status, was increased in smokers in the schizophrenia group, (*t*=3.17, df=18.45, *p*=0.002) but not in the control group (*t*=-0.452, df=18.63, *p*=0.656). No other gene of interest was changed according to smoking status in the control or schizophrenia groups (all: control t<1.18, df=16.86-21.00, *p*>0.05; schizophrenia t<1.30, df=8.43-18.45, *p*>0.05).

When exploring by immune subgroup only, there was no difference between groups with respect to mode of death (χ^2^=1.525, df=2,54, *p*=0.466), although there were more smokers at the time of death in the high immune group (9/10; 90%) compared to the low immune group (18/35; 51%) (χ^2^=4.82, *p*=0.028, *N*=45). Due to small group numbers we are not able to analyze clinical and other categorical factors based on immune/diagnosis group.

**Supplementary Tables**

**Supplementary Table 1.** Human and mouse TaqMan gene expression assays.

| Gene Name | TaqMan Assay |
| --- | --- |
| Allograft inflammatory factor 1 (AIF1) | Hs00741549 |
| Translocator protein (TSPO) | Hs00559362 |
| Cluster of differentiation 68 (CD68) | Hs00154355 |
| HLA-DR | Hs00219575 |
| Interleukin 1β (IL1β) | Hs01555410 |
| Interleukin 6 (IL6) | Hs00174131 |
| Interleukin 6 signal transducer (IL6ST) | Hs01006741 |
| Interleukin 8 (IL8) | Hs00174103 |
| Interleukin 18 (IL18) | Hs01038788 |
| Serpin peptidase inhibitor, Clade A member 3 (SERPINA3) | Hs00153674 |
| Tumour necrosis factor α (TNFα) | Hs99999043 |
| Interleukin 1A | Hs00174092 |
| Interleukin 17RA | Hs01056316 |
| Glial fibrillary acidic protein | Hs00909236 |
| β-actin | Hs99999903 |
| Tata box binding protein (TBP) | Hs004276201 |
| Ubiquitin C (UBC) | Hs00824723 |
| AIF1 | Mm00479862 |
| TNF-α | Mm00443258 |
| SERPINA3 | Mm00776439 |
| IL6 | Mm00446190 |
| IL1β | Mm00434228 |
| IL8^$^ | Mm00441263 |
| IL18 | Mm00434226 |
| TSPO | Mm00437828 |
| GFAP | Mm01253033 |
| 36B4 | Self-designed^+^ |

^$^Expression too low to detect in mouse substantia nigra. **^+^** Forward primer: 5’-AGATGCAGCAGATCCGCAT-3’; reverse primer: 5’-GTTCTTGCCCATCAGCACC-3’; probe: 5’-CGCTCCGAGGGAAGGCCG-3’. Probes and primers for 36B4 have been validated before.^9,22^ Hs. Homo sapien, Mm. Mus Musculus.

**Supplementary References**

1. Purves-Tyson TD, Owens SJ, Rothmond DA, Halliday GM, Double KL, Stevens J *et al.* Putative presynaptic dopamine dysregulation in schizophrenia is supported by molecular evidence from post-mortem human midbrain. *Transl Psychiatry* 2017; **7**(1)**:** e1003.

2. Hardy JA, Wester P, Winblad B, Gezelius C, Bring G, Eriksson A. The patients dying after long terminal phase have acidotic brains; implications for biochemical measurements on autopsy tissue. *J Neural Transm* 1985; **61**(3-4)**:** 253-264.

3. Conley RR, Kelly DL. Management of treatment resistance in schizophrenia. *Biol Psychiatry* 2001; **50**(11)**:** 898-911.

4. Mueller FS, Polesel M, Richetto J, Meyer U, Weber-Stadlbauer U. Mouse modelsv of maternal immune activation: Mind your caging system! *Brain, behavior, and immunity* 2018.

5. Meyer U, Feldon J, Schedlowski M, Yee BK. Towards an immuno-precipitated neurodevelopmental animal model of schizophrenia. *Neurosci Biobehav Rev* 2005; **29**(6)**:** 913-947.

6. Meyer U, Nyffeler M, Engler A, Urwyler A, Schedlowski M, Knuesel I *et al.* The time of prenatal immune challenge determines the specificity of inflammation-mediated brain and behavioral pathology. *J Neurosci* 2006; **26**(18)**:** 4752-4762.

7. Giovanoli S, Notter T, Richetto J, Labouesse MA, Vuillermot S, Riva MA *et al.* Late prenatal immune activation causes hippocampal deficits in the absence of persistent inflammation across aging. *J Neuroinflammation* 2015; **12:** 221.

8. Vuillermot S, Joodmardi E, Perlmann T, Ogren SO, Feldon J, Meyer U. Prenatal immune activation interacts with genetic Nurr1 deficiency in the development of attentional impairments. *J Neurosci* 2012; **32**(2)**:** 436-451.

9. Richetto J, Chesters R, Cattaneo A, Labouesse MA, Gutierrez AMC, Wood TC *et al.* Genome-Wide Transcriptional Profiling and Structural Magnetic Resonance Imaging in the Maternal Immune Activation Model of Neurodevelopmental Disorders. *Cereb Cortex* 2017; **27**(6)**:** 3397-3413.

10. Meyer U, Nyffeler M, Yee BK, Knuesel I, Feldon J. Adult brain and behavioral pathological markers of prenatal immune challenge during early/middle and late fetal development in mice. *Brain, behavior, and immunity* 2008; **22**(4)**:** 469-486.

11. Brown AS, Begg MD, Gravenstein S, Schaefer CA, Wyatt RJ, Bresnahan M *et al.* Serologic evidence of prenatal influenza in the etiology of schizophrenia. *Arch Gen Psychiatry* 2004; **61**(8)**:** 774-780.

12. Canetta SE, Bao Y, Co MD, Ennis FA, Cruz J, Terajima M *et al.* Serological documentation of maternal influenza exposure and bipolar disorder in adult offspring. *Am J Psychiatry* 2014; **171**(5)**:** 557-563.

13. Zorrilla EP. Multiparous species present problems (and possibilities) to developmentalists. *Dev Psychobiol* 1997; **30**(2)**:** 141-150.

14. Notter T, Coughlin JM, Gschwind T, Weber-Stadlbauer U, Wang Y, Kassiou M *et al.* Translational evaluation of translocator protein as a marker of neuroinflammation in schizophrenia. *Mol Psychiatry* 2017.

15. Gundersen HJ, Bagger P, Bendtsen TF, Evans SM, Korbo L, Marcussen N *et al.* The new stereological tools: disector, fractionator, nucleator and point sampled intercepts and their use in pathological research and diagnosis. *APMIS* 1988; **96**(10)**:** 857-881.

16. Howard CV, Reed MG. *Unbiased Stereology*. Bios Scientific Publishers2005.

17. Chiu T, Fang D, Chen J, Wang Y, Jeris C. A robust and scalable clustering algorithm for mixed type attributes in large database environment. *Proceedings of the seventh ACM SIGKDD international conference on Knowledge discovery and data mining* 2001.

18. Bacher J, Wenzig K, Vogler M. SPSS TwoStep Cluster - a first evaluation. *SSOAR* 2004.

19. Fillman SG, Cloonan N, Catts VS, Miller LC, Wong J, McCrossin T *et al.* Increased inflammatory markers identified in the dorsolateral prefrontal cortex of individuals with schizophrenia. *Molecular psychiatry* 2013; **18**(2)**:** 206-214.

20. Steiner J, Bielau H, Brisch R, Danos P, Ullrich O, Mawrin C *et al.* Immunological aspects in the neurobiology of suicide: elevated microglial density in schizophrenia and depression is associated with suicide. *J Psychiatr Res* 2008; **42**(2)**:** 151-157.

21. Furczyk K, Schutova B, Michel TM, Thome J, Buttner A. The neurobiology of suicide - A Review of post-mortem studies. *J Mol Psychiatry* 2013; **1**(1)**:** 2.

22. Richetto J, Calabrese F, Riva MA, Meyer U. Prenatal immune activation induces maturation-dependent alterations in the prefrontal GABAergic transcriptome. *Schizophr Bull* 2014; **40**(2)**:** 351-361.
