# Supplementary material for: Increased levels of midbrain immune-related transcripts in schizophrenia and in murine offspring after maternal immune activation
Source: Mol Psychiatry. 2019 Jun 5;26(3):849–63. doi: 10.1038/s41380-019-0434-0 (PMC7910216; doi:10.1038/s41380-019-0434-0)
Supplement: Supplementary file 2 — Supplementary Figure Legends - Final [file 41380_2019_434_MOESM2_ESM.docx]

**Supplementary Figure Legends**

**Supplementary Figure 1.** Effect of sex on midbrain immune-related transcripts in adult mice prenatally exposed to the viral mimetic, poly(I:C). The scatter plots depict relative mRNA levels separately for male (triangles) and female (circles) offspring and statistical analyses reflect sex effects or sex × treatment interaction (IL1β only), not treatment effects (refer to main manuscript) **(a)** SERPINA3 mRNA was generally higher in males compared to females. There was no interaction between treatment and sex for SERPINA3. **(b)** TNFα was not changed according to sex and there was no interaction between treatment and sex. **(c)** IL6 mRNA was generally increased in male offspring with no interaction between sex and treatment. **(d)** IL18 mRNA was different according to sex but there was no sex and treatment interaction. **(e)** IL1β mRNA was not changed according to sex, but showed a treatment and sex interaction with IL1β mRNA only increasing in female poly(I:C) offspring. **p*<0.05, ***p*<0.01, *****p*<0.0001. Data is mean ± SEM

**Supplementary Figure 2.** Cytokine proteins in the midbrain of adult mice prenatally exposed to the viral mimetic, poly(I:C), and vehicle-exposed control mice. The scatter plots depict **(a)** TNFα, **(b)** IL6 and **(c)** IL1β protein levels (expressed in pg/mg total protein) by prenatal treatment group (poly(I:C) versus vehicle controls). There were sex effects or sex × treatment interactions on **(d)** TNFα, **(e)** IL6 or **(h)** IL1β protein levels. Data are mean ± SEM. *p<0.05, **p<0.01.

**Supplementary Figure 3**. Effect of sex on midbrain microglial and astrocyte marker transcripts and microglial cell densities in adult mice prenatally exposed to the viral mimetic, poly(I:C). The scatter plots depict relative mRNA levels separately for male (triangles) and female (circles) offspring. Prenatal poly(I:C) exposure and sex did not modify **(a)** AIF1 **(b)** TSPO or **(c)** GFAP mRNA levels, or **(d)** AIF1+ microglial cell density, **(e)** CD68+ microglial cell density or **(f)** GFAP+ astrocytic cell density. There were no treatment × sex interactions in any of these measures. Data is mean ± SEM

**Supplementary Figure 4.** Changes in the immune-related transcripts used to generate the immune clusters in midbrain from schizophrenia (SCZ) and control cases and from adult mice prenatally exposed to the viral mimetic, poly(I:C), after stratification in to “high” and “low” immune subgroups. **(a)** SERPINA3, **(b)** TNFα, **(c)** IL6 and **(d)** IL1β mRNAs were all increased in the SCZ/high immune compared to the SCZ/low immune subgroup and compared to the control group. **(e)** IL8 and **(f)** IL18 mRNAs were unchanged based on diagnosis/immune status. **(g)** IL6ST mRNA was increased in both SCZ/high and SCZ/low immune subgroups compared to the control group. **(h)** SERPINA3, **(i)** TNFα, **(j)** IL6 and **(k)** IL1β mRNAs were all increased in the poly(IC)/high immune subgroup compared to the poly(IC)/low immune and vehicle/low immune subgroups. **(l)** IL18 mRNA was unchanged based on prenatal treatment/immune status. The vehicle/high immune group is shown on graph for visual comparison but was excluded from the analysis. Data are mean ± SEM. **p*<0.05, ***p*<0.01, ****p*<0.001, *****p*<0.0001.

**Supplementary Figure 5.** Treatment of hCMEC/D3 endothelial cells with antipsychotics. **(a)** IL1β mRNA was reduced by risperidone (RIS) treatment but was not changed by clozapine (CZP) or haloperidol (HAL) treatment compared to vehicle treatment (5% fetal bovine serum, FBS) (n=7/8 per group). IL6 **(b)** and SERPINA3 **(c)** mRNAs were not changed by any antipsychotic treatment compared to vehicle-treated control. Data presented as mean ± SEM. **p*<0.05.
