# Supplementary figures and images for: Increased levels of midbrain immune-related transcripts in schizophrenia and in murine offspring after maternal immune activation
Source: Mol Psychiatry. 2019 Jun 5;26(3):849–63. doi: 10.1038/s41380-019-0434-0 (PMC7910216; doi:10.1038/s41380-019-0434-0)

# Supplementary Figure 1

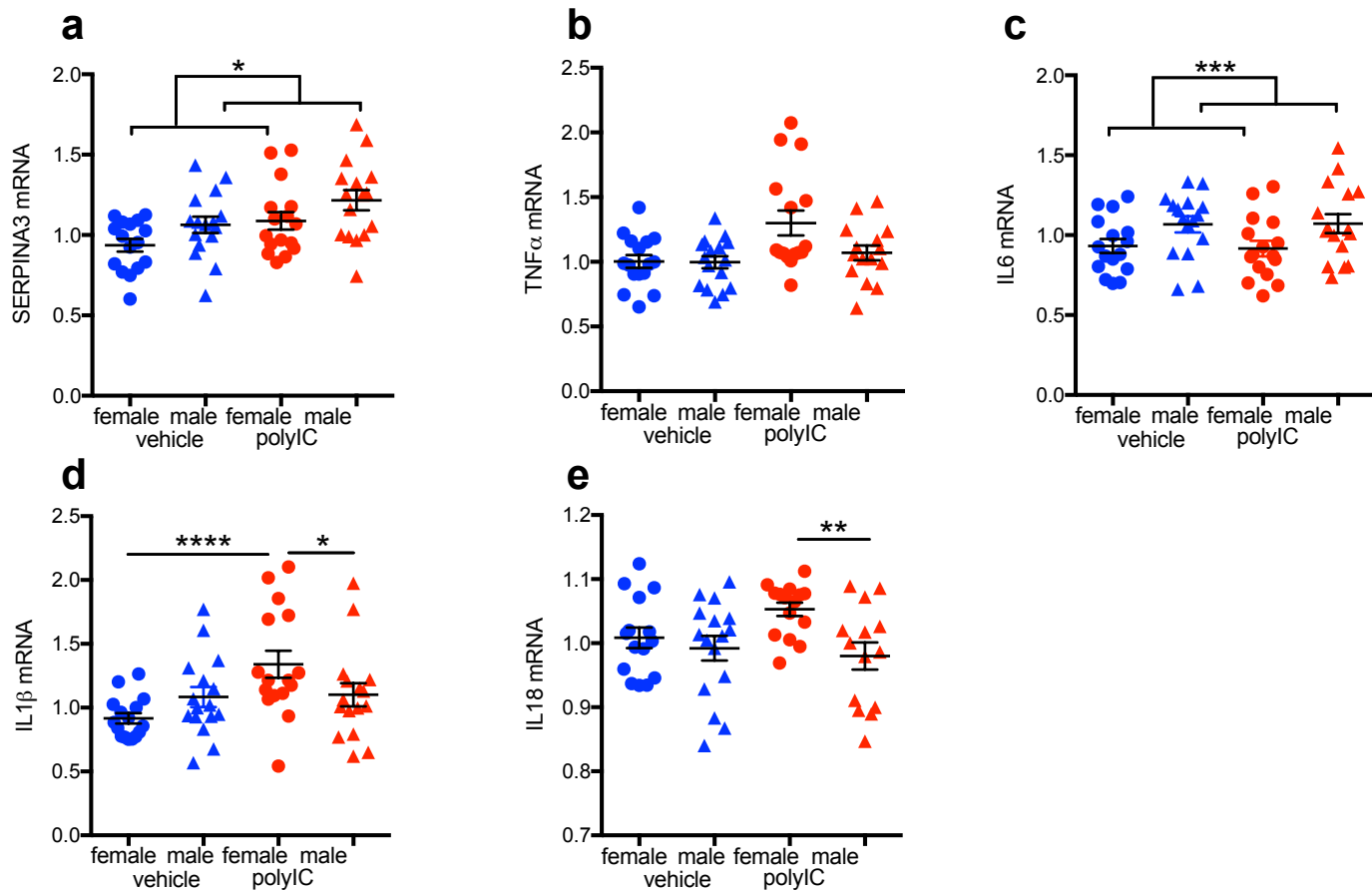

Supplement: Supplementary file 3 — Supplementary Figure 1 [file 41380_2019_434_MOESM3_ESM.pdf]

# Supplementary Figure 2

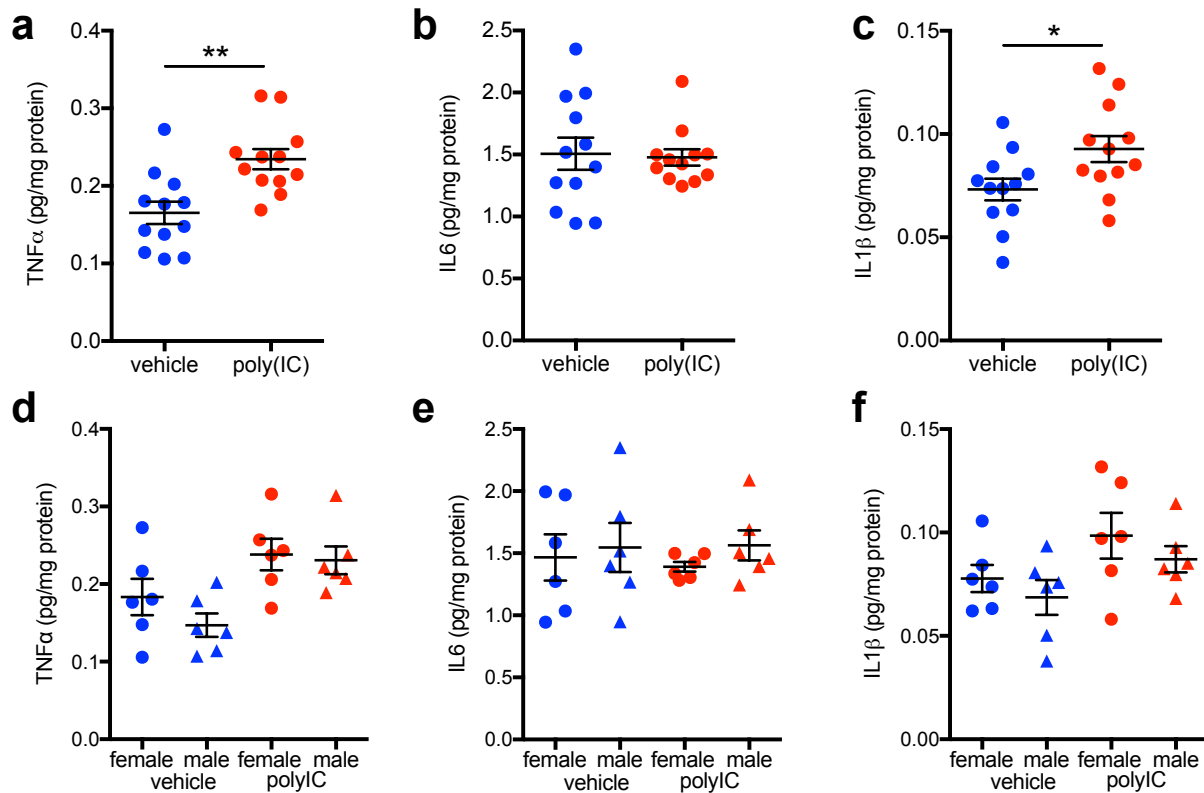

Supplement: Supplementary file 4 — Supplementary Figure 2 [file 41380_2019_434_MOESM4_ESM.pdf]

# Supplementary Figure 3

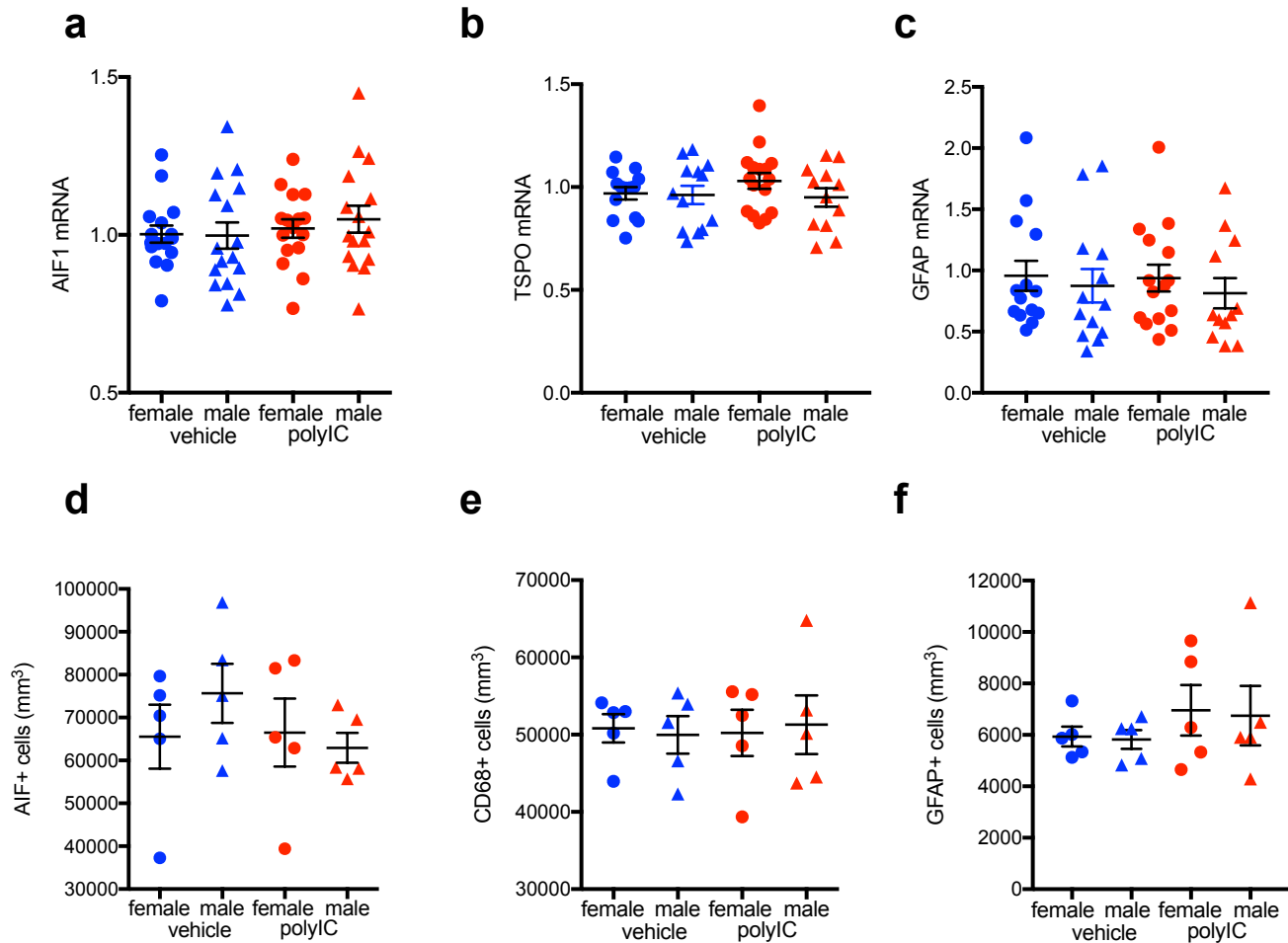

Supplement: Supplementary file 5 — Supplementary Figure 3 [file 41380_2019_434_MOESM5_ESM.pdf]

# Supplementary Figure 4

## Human

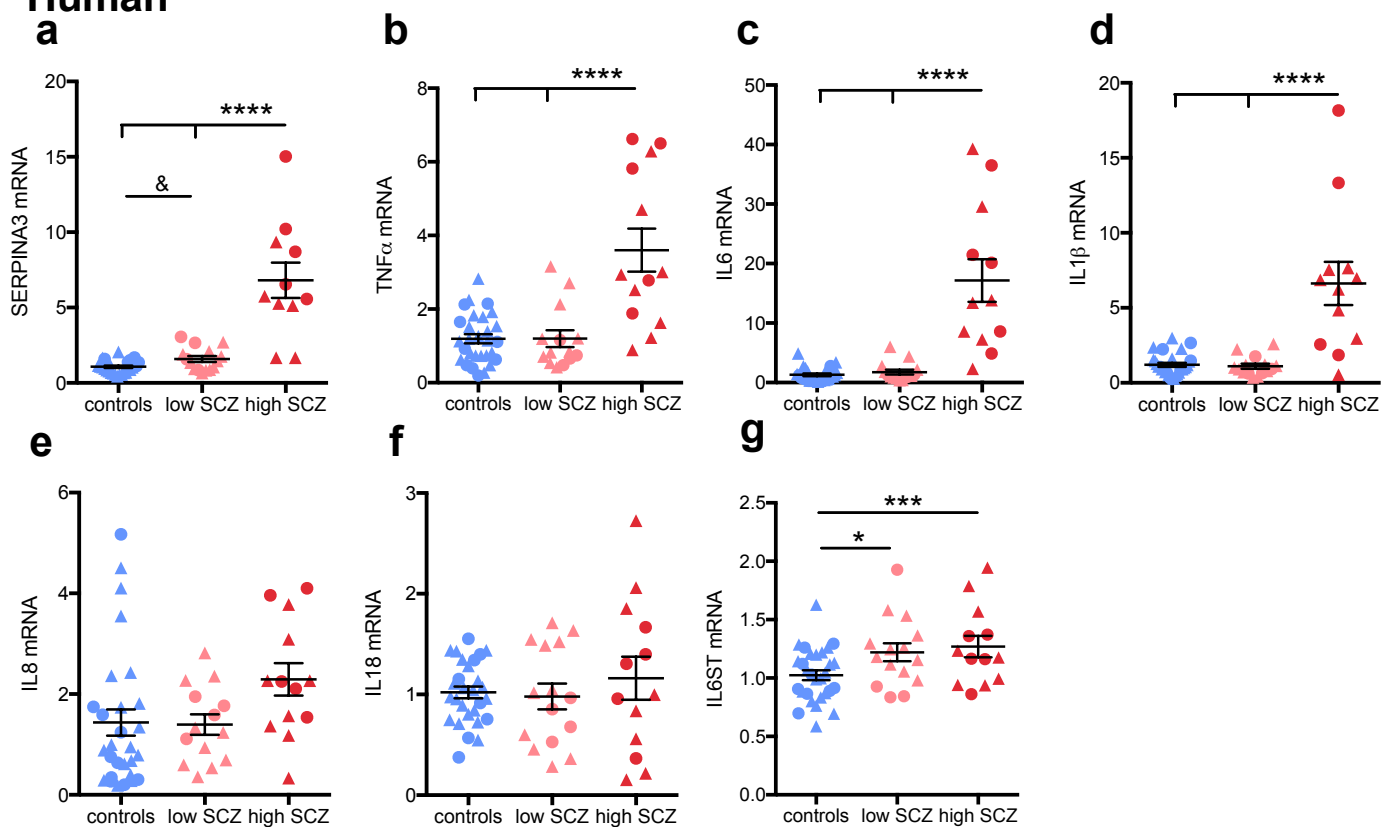

## Mouse

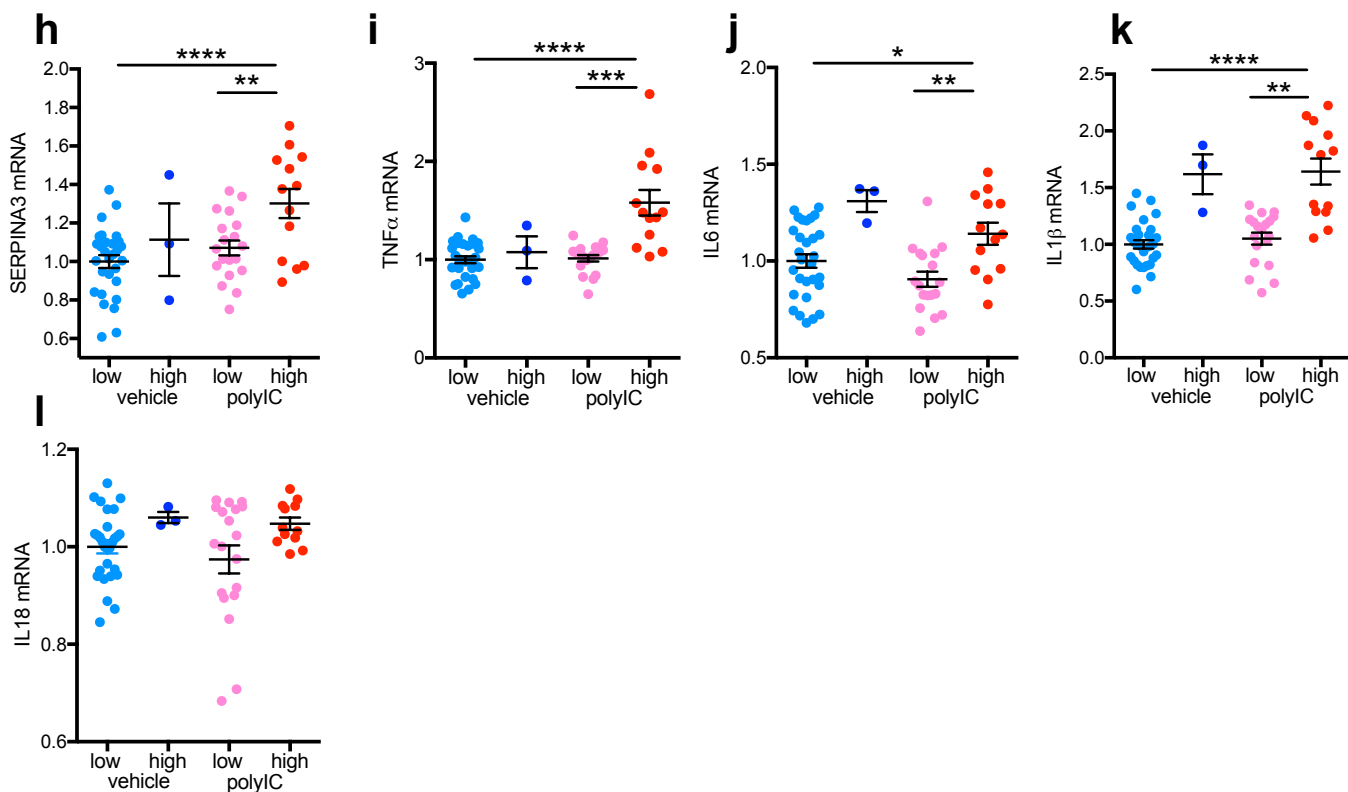

Supplement: Supplementary file 6 — Supplementary Figure 4 [file 41380_2019_434_MOESM6_ESM.pdf]

# Supplementary Figure 5

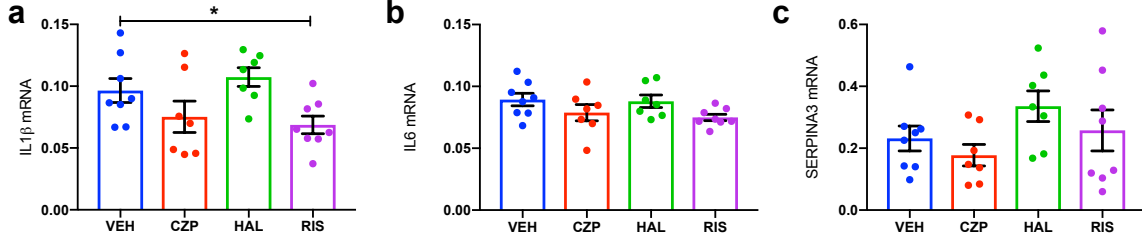

Supplement: Supplementary file 7 — Supplementary Figure 5 [file 41380_2019_434_MOESM7_ESM.pdf]
